# The representation of women on Australian clinical practice guideline panels, 2010–2020

**DOI:** 10.5694/mja2.51831

**Published:** 2023-01-04

**Authors:** Anna Shalit, Lauren Vallely, Renae Nguyen, Meghan Bohren, Agnes Wilson, Caroline SE Homer, Joshua Vogel

**Affiliations:** ^1^ The Burnet Institute Melbourne VIC; ^2^ Melbourne School of Population and Global Health University of Melbourne Melbourne VIC; ^3^ Hereco Sydney NSW; ^4^ Cochrane Australia Monash University Melbourne VIC

**Keywords:** Physicians, women, Guidelines as topic, Public health, Health policy

## Abstract

**Objectives:**

To assess the composition by gender of Australian clinical practice guideline development panels; to explore guideline development‐related factors that influence the composition of panels.

**Design, setting, participants:**

Survey of clinical guidelines published in Australia during 2010–2020 that observed the 2016 *NHMRC Standards for Guidelines*, identified (June 2021) in the NHMRC Clinical Practice Guideline Portal or by searching the Guideline International Network guidelines library, the Trip medical database, and PubMed. The gender of contributors to guideline development was inferred from gendered titles (guideline documents) or pronouns (online biographies).

**Main outcome measures:**

The overall proportion of guideline panel members — the guideline contributors who formally considered evidence and formulated recommendations (ie, guideline panel chairs and members) — who were women.

**Results:**

Of 406 eligible guidelines, 335 listed the names of people who contributed to their development (82%). Of 7472 named contributors (including 511 guideline panel chairs [6.8%] and 5039 guideline panel members [67.4%]), 3514 were men (47.0%), 3345 were women (44.8%), and gender could not be determined for 612 (8.2%). A total of 215 guideline panel chairs were women (42.1%), 280 were men (54.8%); 2566 guideline panel members were men (50.9%), 2071 were women (41.1%). The proportion of female guideline panel members was smaller than 40% for 179 guidelines (53%) and larger than 60% for 71 guidelines (21%). The median guideline proportion of female panel members was smaller than 50% for all but two years (2017, 2018).

**Conclusions:**

The representation of women in health leadership roles in Australia does not reflect their level of participation in the health care workforce. In particular, clinical guideline development bodies should develop transparent policies for increasing the participation of women in guideline development panels.

**The known:** Despite the growing proportion of women in health care professions, men predominate in most leadership roles, including clinical practice guideline development. The gender composition of Australian clinical guideline panels has not been reported.

**The new:** For 179 of 335 high quality Australian clinical guidelines published during 2010–2020 (53%), the proportion of women in guideline development groups was smaller than 40%; 280 of 511 chairs were men (55%).

**The implications:** The gender balance of Australian guideline panels should be improved. Gender‐based inequity in health care is a complex problem, but must be overcome to ensure high quality and equitable health care for all.

Although in some countries 75% of the health workforce are women, leadership roles in medicine are often dominated by men.^1^ In Australia, there were 2.9 registered female health professionals for each male professional in 2020, but most medical and dental practitioners were men.^2^ In 2019, 45% of public hospital board chairs in Australia were women (but only 13% in New South Wales).^3^ Women comprise increasingly large proportions of National Health and Medical Research Council (NHMRC) funding recipients, but in 2021 received only 38.5% of total Investigator Grant funding (the largest funding scheme), despite structural priority funding measures.^4^ Gender inequities in support have negative consequences for research, health policy development, and health outcomes.^5^ At the health leadership level, they harm morale, lead to the loss of critical skills, and have negative impacts on health care services;^6^, ^7^, ^8^ redressing gender‐based inequity could improve scientific knowledge production, medical practice, and patient outcomes.^9^

Guideline development bodies overseas have policies for ensuring gender equity in guideline development, a critical area of health care leadership. The World Health Organization Guideline Review Committee recommends that guideline development groups be “balanced in terms of gender and geography”.^10^ In Australia, the NHMRC recommends that guideline development groups be transparently reported and representative of the patients to whom the guideline will be applied.^11^, ^12^

Nevertheless, substantial gender imbalances in guideline panels have been reported.^13^, ^14^, ^15^, ^16^, ^17^, ^18^, ^19^, ^20^, ^21^, ^22^, ^23^, ^24^ In a study that included 454 clinical guidelines published during 2012–2017, 38% of guideline authors were women;^18^ similarly, fewer than 40% of panel members were women for about one‐third of WHO guidelines (2008–2018).^13^ As corresponding information has not been published for Australia, we examined the composition by gender of Australian clinical practice guideline panels, both overall and by health topic, and also examined guideline development‐related factors that might influence panel composition.

## Methods

We registered our study protocol with the Open Science Framework (12 August 2021; updated 18 August 2021; osf.io/n594f).

### Guideline selection

We selected national guidelines with recommendations for health professionals in Australia published during 2010–2020 that satisfied the 2016 *NHMRC Standards for Guidelines*; these included being publicly accessible, being based on systematic reviews of evidence, development by a professional organisation, and containing a statement on conflicts of interest.^12^

We identified guidelines in two ways. First, the NHMRC Research Translation Office (Clinical Practice Guidelines) provided a spreadsheet of guidelines indexed in their database (the NHMRC clinical practice guideline portal) during 1 January 2010 – 31 December 2020. We retrieved and screened the listed guidelines for eligibility for our analysis.

Second, assisted by an information specialist, we searched the Guideline International Network guidelines library (https://g‐i‐n.net/international‐guidelines‐library), the Trip medical database (“guideline” filter function in advanced version; https://www.tripdatabase.com), and PubMed (24–26 June 2021; Supporting Information, supplementary methods). Citations were imported into Covidence (https://www.covidence.org), duplicates removed, and the cited items independently screened by two authors (AS, LV).

Finally, eligible guidelines from the two sources were then merged into a single list and duplicates removed.

### Data extraction and analysis

We used a standardised form for data extraction. For each guideline we extracted the guideline title, publication year, publishing organisation, funding source, use of Grading of Recommendations, Assessment, Development and Evaluation (GRADE) methodology, NHMRC guideline approval status, and health topic (ie, primary specialty of the publishing organisation).

We then extracted the name, role, and gender of all named contributors to the guideline development process, grouped into three role categories: guideline panel chair, guideline panel member, and other contributors (steering committee chair, steering committee member, advisory committee chair, advisory committee member, technical project leadership role, technical project team member). The focus of our analysis was the guideline panel; that is, the guideline development group members who formally considered evidence and formulated recommendations (guideline panel chairs and members).

The WHO defines sex as biologically determined and gender as socially defined.^25^ For our analysis, we examined gender (categories: man, woman, other, unknown), based on information in the guideline and gendered titles (eg, Miss, Mr, Mrs, Ms). When the guideline did not yield this information, we searched online for professional biographies (eg, institutional, professional association websites) for pronouns or gendered titles as proxies for gender; when this information was unavailable, gender was classified as “unknown”. Neither names nor photos were used at any stage for determining gender. Gender was extracted by one author (AS) and independently reviewed by another (LV or RN), and the data were de‐identified for analysis and reporting.

The primary outcome was the overall proportion of guideline panel members who were women; a sensitivity analysis excluded guidelines with fewer than ten guideline members (excluding chairs). We assigned each guideline to one of three categories by the proportion of women (fewer than 40%; 40–60%; more than 60%). These categories reflected the 2019 Australian Medical Association gender diversity target of at least 40% representation for both men and women^26^ and the Science in Australia Gender Equity definition of gender balance.^27^ The statistical significance of differences in the proportion of guidelines for which fewer than 40% of guideline panel members were women by NHMRC approval, use of GRADE methodology, or source of funding was assessed in χ^2^ tests; *P* < 0.05 was deemed statistically significant. A further planned analysis compared the proportion of women in guideline panels from the three health topics with the largest number of guidelines (cancer, cardiology, nephrology), as well as in paediatric medicine and women's health. Statistical analyses were undertaken in Stata 16.

### Ethics approval

We did not seek ethics approval for our analysis of publicly available information.

## Results

We initially selected 406 guidelines for our analysis (Supporting Information, table 1), but 71 guidelines (18%) that did not identify guideline panel members were excluded (Box 1). Of the 335 included guidelines in 35 health topics (Supporting Information, table 2), 78 reported using GRADE methodology (23%) and 59 were approved by the NHMRC (18%). The NHMRC or federal government funded 74 guidelines (22%), other sources funded 89 guidelines (27%); 14 guidelines reported they had received no financial support (4%), and 158 guidelines did not describe funding (47%).

The 7472 contributors named in the 335 guidelines (median per guideline, 12 people; interquartile range, 6–22 people) included 3514 men (47.0%) and 3345 women (44.8%); gender could not be determined for 612 people (8.2%), and one person used they/them as pronouns in their online professional profile and did not specify a gender identity.

Box 1 **Selection of Australian clinical guidelines meeting National Health and Medical Research Council standards published during 2010–2020 for our analysis**

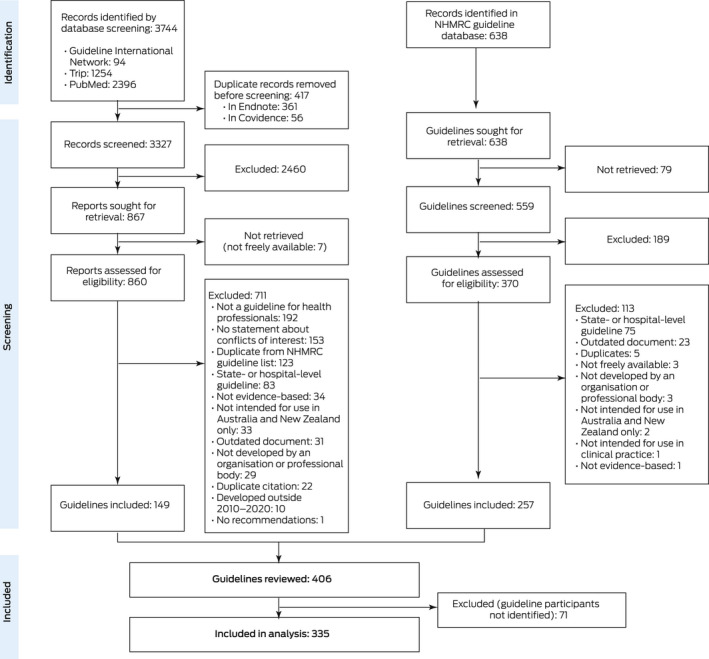

### Guideline panel chairs

Of the 511 guideline panel chairs (6.8% of named guideline contributors), 280 were men (54.8%) and 215 were women (42.1%); gender could not be determined for 16 people (3.1%). The overall proportion of chairs was larger for men than women in all years apart from 2017 (38 of 61, 62% women) (Supporting Information, table 3).

### Guideline panel members

The 5039 guideline panel members (67.4% of named guideline contributors) included 2566 men (50.9%) and 2071 women (41.1%); gender could not be determined for 402 people (8.0%). The proportion of women was smaller than 40% for 179 guidelines (53%; including 36 with fewer than 10% women [11%]), 40–60% for 85 guidelines (25%), and larger than 60% for 71 guidelines (21%). The overall proportion of guideline panel members was larger for men than women in all years apart from 2011 (288 of 572, 50% women) and 2017 (297 of 536, 55% women) (Supporting Information, table 3). The median proportion of women by year was below 50% each year, with the exceptions of 2017 and 2018 (Box 2). In the sensitivity analysis (199 guidelines with ten or more guideline panel members), the proportion of women was smaller than 40% for 103 (52%), 40–60% for 55 (28%), and larger than 60% for 41 guidelines (21%).

Box 2 **Proportion of Australian guideline panel members who were women, for 335 guidelines published during 2010–2020**

**Figure:**
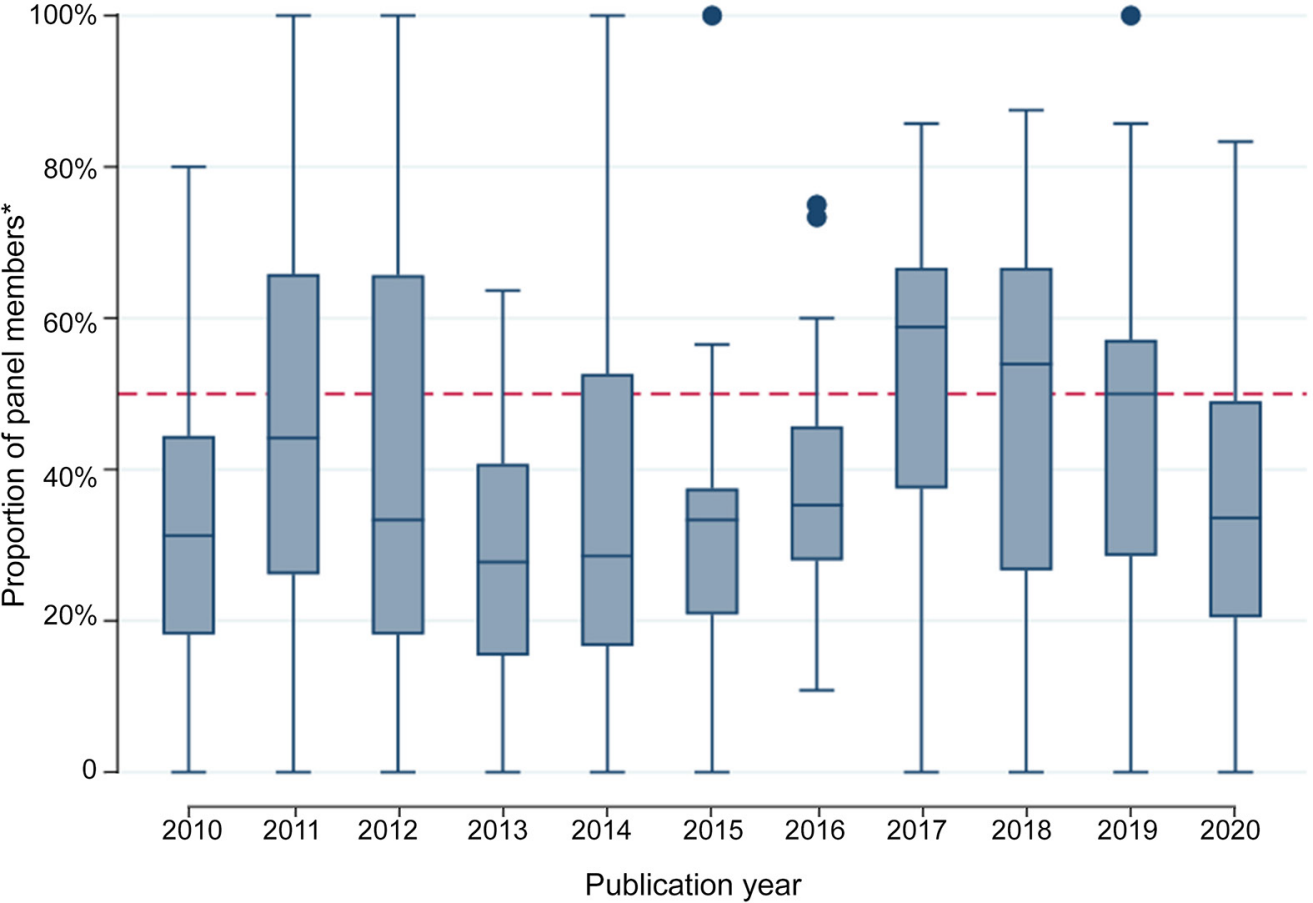
* Does not include guideline development group chairs. Each box represents the interquartile range of values (with the median indicated by a horizontal line); whiskers indicate maximum values, circles outlier values excluded from the median calculation. The number of included guidelines by year is included in the Supporting Information, table 3.

### Guideline panel members: by guideline characteristic

Women comprised fewer than 40% of guideline panel members for 17 of 59 NHMRC‐approved guidelines (29%) and 162 of 276 guidelines without NHMRC approval (59%; *P* < 0.001). Women comprised fewer than 40% of guideline panel members for 32 of 78 guidelines that used GRADE methodology (41%) and 147 of 257 guidelines that did not (57%; *P* = 0.043). As 216 guidelines did not report their funding source, we did not assess whether the proportion of women guideline panel members differed by this characteristic.

In our additional analysis, the proportion of guideline panel members who were women was below 40% for 22 of 50 cancer guidelines (44%), 31 of 39 cardiology guidelines (80%), 18 of 27 nephrology guidelines (67%), four of 13 paediatric medicine guidelines (31%), and two of 17 women's health guidelines (12%) (Box 3; Supporting Information, table 2).

Box 3 Proportion of Australian guideline panel members* who were women, for guidelines published during 2010–2020, by guideline health topic

**Figure:**
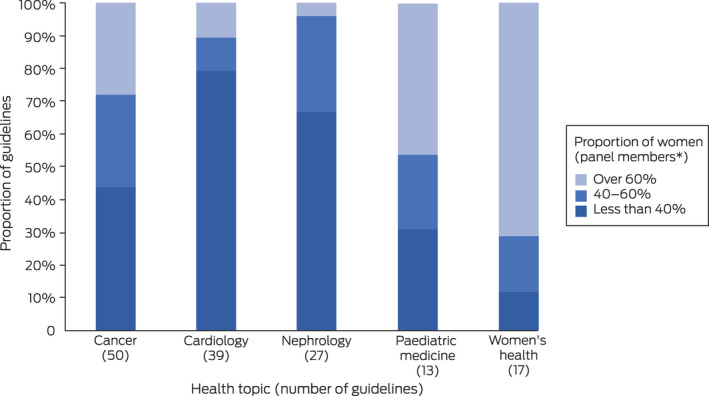
* Does not include guideline development group chairs. The proportions for other health topics are included in the Supporting Information, table 2.

## Discussion

We report the first analysis of the composition of Australian clinical practice guideline panels by gender. The overall proportion of women as contributors to guideline development (all roles) was 44.8%, as guideline panel members 41.1%, and as guideline panel chairs 42.1%. The proportion of female guideline panel members was smaller than 40% for 179 of 335 guidelines (53%); the proportion was smaller for NHMRC‐approved guidelines (17 of 59, 29%), and larger in some health areas (eg, cardiology, 80%; nephrology, 67%) than others (eg, women's health, 12%; paediatric medicine, 31%).

Our findings are consistent with those of other reports of underrepresentation of women on guideline panels in various countries,^13^, ^14^, ^15^, ^16^, ^17^, ^18^, ^19^, ^20^, ^21^, ^22^, ^23^, ^24^ particularly in leadership roles such as panel chairs.^17^, ^19^, ^22^ The high proportion of cardiology guidelines with low female guideline panel member proportions is consistent with American and Canadian findings.^16^, ^24^ This is of particular concern given the burden of cardiovascular disease in Australia and recognised gender‐based differences in its diagnosis and treatment.^28^, ^29^, ^30^ In contrast, the proportions of women were larger for guideline panels in areas in which women are traditionally more prominent, such as women's and children's health.

Although the proportion of female medical doctors is rising in Australia^31^ and gender diversity is increasingly promoted in health care,^32^ underrepresentation of women in health leadership positions is often attributed to factors such as gender‐based discrimination, carer responsibilities, and maternity leave.^33^ As unconscious biases during the informal process of guideline panel member selection can contribute to unequal representation,^17^, ^18^, ^19^ more structured, transparent selection processes could help correct imbalances.

Workplace culture, quality of care, and all patients could benefit from the increased creativity and productivity associated with gender balance.^32^, ^34^ For example, scientific publications with female authors are more likely to take gender and sex into consideration, increasing the potential for findings that lead to equitable health care.^35^ Policymakers and guideline developers should consider the many benefits of equitable gender representation^5^ when convening guideline panels, and the effect of gender balance on guideline quality should be investigated.

Despite NHMRC recommendations,^11^, ^12^ 71 of 405 guidelines otherwise eligible for our analysis (18%) did not report guideline contributors, and 216 of 335 included guidelines did not report how they were funded (64%). We therefore recommend that the listing of guideline panel members (including their gender, with consent) and the description of panel recruitment processes and funding sources be required for Australian guidelines.

Prescriptive recommendations and quotas for gender equity in guideline development could improve balance.^13^ The lower proportion of NHMRC‐approved guidelines with fewer than 40% female guideline panel members is consistent with this view. Organisations that develop or endorse guidelines should adopt effective policies for gender balance on their guideline panels and regularly monitor progress toward this target.

### Limitations

The gender of guideline panel members was not consistently reported. Other investigators have inferred gender from names and photos,^17^, ^18^, ^19^, ^23^ but, as this approach is susceptible to error and bias, we instead inferred gender from pronouns and gendered titles. This may have reduced the likelihood of error, but we could not determine gender for 8.2% of guideline panel members. We could not assess whether gender identity was different at the time of guideline publication and at data collection. Given the large variability in size of guideline panels, our findings might be skewed by those with small or very large panels. However, a sensitivity analysis excluding guidelines with fewer than ten panel members yielded similar results to the main analysis. We did not assess the proportions of women in guideline commissioning organisations, and this was therefore not compared with the number of women on their guideline panels. Patient and community involvement in guideline development, recommended by the NHMRC, was not assessed in our study.^12^ The intersection of gender with other socio‐cultural factors (eg, ethnic or social background) is critical for discussions of inequity in health leadership,^36^, ^37^ but such factors are not typically reported by guidelines, nor can they be easily assessed retrospectively.

### Conclusions

Clinical practice guidelines are a key component of evidence‐based health care, and guideline development is an important part of health care leadership. We found that women are underrepresented in Australian guideline development groups. Australian guideline development organisations should revise the manner in which they recruit guideline development panels in order to secure gender balance; particularly important is transparent reporting and regular auditing of panel composition. Gender bias in health care is a complex and multifactorial problem, but it must be overcome to ensure high quality and equitable health care.

## Open access

Open access publishing facilitated by Monash University, as part of the Wiley – Monash University agreement via the Council of Australian University Librarians.

## Competing interests

Meghan Bohren, Caroline Homer, Agnes Wilson, and Joshua Vogel were involved in the development of several guidelines included in our study. Caroline Homer chairs the NHMRC; Joshua Vogel is a member of the NHMRC Research Committee, but this played no role in access to the guidelines or in the analysis. These findings are those of the authors and do not necessarily reflect the views of the NHMRC.

## Supporting information


**Table S1** Full list of the clinical guidelines assessed
**Table S2**. Overall proportions of guideline panel members who were women in 335 guidelines, by health topic
**Table S3**. The 335 included guidelines: numbers by year of publication, and numbers of female guideline panel chairs and members
