# Supplementary material for: The representation of women on Australian clinical practice guideline panels, 2010–2020
Source: Med J Aust. 2023 Jan 4;218(2):84–8. doi: 10.5694/mja2.51831 (PMC10953318; doi:10.5694/mja2.51831)
Supplement: Supplementary file 1 — Table S1 Full list of the clinical guidelines assessed Table S2. Overall proportions of guideline panel members who were women in 335 guidelines, by health topic Table S3. The 335 included guidelines: numbers by year of publication, and numbers of female guideline panel chairs and members [file MJA2-218-84-s001.pdf]

The Medical Journal of Australia

Supporting Information

Supplementary methods and results

This appendix was part of the submitted manuscript and has been peer reviewed.
It is posted as supplied by the authors.

Appendix to: Shalit A, Vallely L, Nguyen R, et al. The representation of women on Australian clinical practice guideline panels, 2010–2020. *Med J Aust* 2023; doi: 10.5694/mja2.51831.

Supplementary methods: search strategy

PubMed search strategy

Search number	Query	Filters	Search Details
4	#1 AND #2	from 2010 - 2020	("Australia*"[MeSH Terms] AND "guideline*"[MeSH Terms]) AND (2010:2021[pdat])
3	#1 AND #2		"Australia*"[MeSH Terms] AND "guideline*"[MeSH Terms]
2	Guideline*[MESH]		"guideline*"[MeSH Terms]
1	Australia*[MESH]		"Australia*"[MeSH Terms]

GIN library search strategy

Search number	Query	Filters	Search Details
1	*No search terms entered*	Publication year: 2010 – 2020<br>Countries of application: Australia and New Zealand	

TRIP database search strategy

Search number	Query	Filters	Search Details
1	*All entries from 2010-2020*	“Guidelines”<br>“Aus & NZ”	

Table 1. Full list of the clinical guidelines assessed

Guideline name	URL	Year	Health topic	NHMRC-approved	GRADE	Funding source
**Ten or more guideline panel members and chairs**						
**1. Australian evidence-based clinical guidelines for diabetes**	https://files.magicapp.org/guideline/3b03964b-0a09-4d3f-a5da-c4e4ad174bfa/published_guideline_4496-1_2.pdf	2020	Endocrinology	Yes	Yes	Federal government
**2. Australian guidelines for the clinical care of people with covid-19**	https://files.magicapp.org/guideline/5385329d-4e37-43d4-aa99-3ae393bfa59f/published_guideline_5360-40_0.pdf	2020	Infectious diseases	Yes	Yes	Federal government
**3. Clinical practice guideline for the management of communication and swallowing in children diagnosed with childhood brain tumour or leukaemia**	https://www.mcri.edu.au/sites/default/files/media/documents/tbi_guideline.pdf	2020	Paediatrics	Yes	Yes	Other
**4. Australian guidelines for the prevention and treatment of acute stress disorder, posttraumatic stress disorder, and complex posttraumatic stress disorder**	https://files.magicapp.org/guideline/357a1e08-05d4-44b1-89dd-e15a971b3bbf/published_guideline_4672-5_6.pdf https://www.phoenixAustralia.org/wp-content/uploads/2020/07/Administrative-report-1.pdf	2020	Psychiatry	Yes	Yes	Federal government
**5. Clinical practice guidelines for keratinocyte cancer**	https://wiki.cancer.org.au/Australiawiki/images/e/ed/KC_Summary_of_recommendations_JUN19.pdf	2019	Cancer	Yes	Yes	Federal government
**6. Clinical practice guidelines for surveillance colonoscopy**	https://wiki.cancer.org.au/Australia/Guidelines:Colorectal_cancer/Colonoscopy_surveillance	2019	Cancer	Yes	Yes	Federal government
**7. Clinical guideline for the diagnosis and management of work-related mental health conditions in general practice**	https://www.monash.edu/__data/assets/pdf_file/0004/1696702/Work-Related-Mental-Health-Clinical-Guideline-for-GPs_Digital_Update-2019.09.10.pdf	2019	General Practice	Yes	Yes	NHMRC
**8. Guideline for the management of knee and hip osteoarthritis**	https://www.racgp.org.au/download/Documents/Guidelines/Musculoskeletal/guideline-for-the-management-of-knee-and-hip-oa-2nd-edition.pdf	2018	General Practice	Yes	Yes	Other
**9. Australian immunisation handbook (2018)**	https://www.health.gov.au/resources/publications/the-Australian-immunisation-handbook	2018	Immunology	Yes	Yes	Federal government
**10. A national guideline for the assessment and diagnosis of autism spectrum disorders in Australia**	https://www.autismcrc.com.au/access/sites/default/files/resources/National_Guideline_Summary_and_Recommendations.pdf	2018	Paediatrics	Yes	Yes	Other
**11. Clinical practice guidelines: pregnancy care: 2018 edition**	https://ranzcog.edu.au/RANZCOG_SITE/media/RANZCOG-MEDIA/Women's%20Health/Patient%20information/Pregnancy-Care-Guidelines.pdf	2018	Women’s Health	Yes	Yes	Federal government
**12. Evidence-based clinical practice guideline for deprescribing cholinesterase inhibitors and memantine**	https://cdpc.sydney.edu.au/wp-content/uploads/2019/06/deprescribing-recommendations.pdf	2018	Geriatrics	Yes	Yes	NHMRC
**13. RANZCOG home births**	https://ranzcog.edu.au/RANZCOG_SITE/media/RANZCOG-MEDIA/Women%27s%20Health/Statement%20and%20guidelines/Clinical-Obstetrics/Home-Births-(C-Obs-2)-Review-July-17.pdf?ext=.pdf	2017	Women’s Health	Yes	Yes	Other

Guideline name	URL	Year	Health topic	NHMRC-approved	GRADE	Funding source
**14. Clinical practice guideline for the care of women with decreased fetal movements**	https://ranzcog.edu.au/RANZCOG_SITE/media/RANZCOG-MEDIA/Women%27s%20Health/Statement%20and%20guidelines/Clinical-Obstetrics/Home-Births-(C-Obs-2)-Review-July-17.pdf?ext=.pdf	2017	Women's Health	No	Yes	Other
**15. Clinical practice guidelines for the prevention, early detection and management of colorectal cancer**	https://wiki.cancer.org.au/Australiawiki/images/e/ed/Colorectal_cancer_guidelines_short_form.pdf	2017	Cancer	Yes	Yes	Federal government
**16. Mental health care in the perinatal period: Australian clinical practice guideline**	https://cope.org.au/wp-content/uploads/2017/10/Final-COPE-Perinatal-Mental-Health-Guideline.pdf	2017	Women's Health	Yes	Yes	Federal government
**17. Clinical guidelines for stroke management 2017**	https://informme.org.au/en/Guidelines/Clinical-Guidelines-for-Stroke-Management	2017	Neurology	Yes	Yes	Federal government
**18. Nutrition guidelines for cystic fibrosis in Australia and New Zealand**	https://www.thoracic.org.au/documents/item/1045	2017	Respiratory	Yes	Yes	Other
**19. Australia and New Zealand pulmonary rehabilitation guidelines**	https://lungfoundation.com.au/wp-content/uploads/2018/09/Book-Australia-and-New-Zealand-Pulmonary-Rehabilitation-Guidelines-Feb2017.pdf	2017	Respiratory	No	Yes	Other
**20. Clinical practice guideline for the management of communication and swallowing disorders following paediatric traumatic brain injury**	https://www.mcri.edu.au/sites/default/files/media/documents/tbi_guideline.pdf	2017	Paediatrics	Yes	Yes	NHMRC
**21. RANZCP clinical practice guideline for the management of deliberate self-harm**	https://www.ranzcp.org/files/resources/college_statements/clinician/cpg/deliberate-self-harm-cpg.aspx	2016	Psychiatry	No	Yes	Other
**22. Clinical practice guidelines PSA testing and early management of test-detected prostate cancer**	https://wiki.cancer.org.au/Australiawiki/images/1/1b/PSA_Testing_and_Early_Management_of_Test-detected_Prostate_Cancer_-_Clinical_practice_guidelines_-_Nov15.pdf	2016	Cancer	Yes	Yes	Other
**23. Australian asthma handbook**	https://extranet.who.int/ncdccs/Data/AUS_D1_AsthmaManagementV1-2-QRG.pdf	2016	Respiratory	No	Yes	Other
**24. Patient blood management guidelines: module 6 - neonatal and paediatrics**	https://www.blood.gov.au/system/files/14523_NBA-Module-6-Neonat_Paediatrics_internals_5_updated_14_May_2020.pdf	2016	Haematology	Yes	Yes	Other
**25. Clinical practice guidelines and principles of care for people with dementia**	https://cdpc.sydney.edu.au/wp-content/uploads/2019/06/CDPC-Dementia-Guidelines_WEB.pdf	2016	Geriatrics	Yes	Yes	NHMRC
**26. National evidence based guideline on secondary prevention of cardiovascular disease in type 2 diabetes**	https://extranet.who.int/ncdccs/Data/AUS_D1_National%20Evidence-based%20Guideline%20on%20Secondary%20Prevention%20of%20Cardiovascu....pdf	2015	Endocrinology	Yes	Yes	Federal government
**27. Patient blood management guidelines: module 5 - obstetrics and maternity**	https://www.blood.gov.au/system/files/documents/20180426-Module5-WEB.pdf	2015	Haematology	Yes	Yes	Other
**28. Clinical practice guidelines: pregnancy care: 2011 edition**	https://www.health.gov.au/sites/default/files/documents/2021/02/pregnancy-care-guidelines-pregnancy-care-guidelines.pdf	2011	Women's Health	Yes	Yes	Federal government
**29. Clinical practice guidelines: pregnancy care: 2014 edition**	https://www.health.gov.au/sites/default/files/documents/2021/02/pregnancy-care-guidelines-pregnancy-care-guidelines.pdf	2014	Women's Health	Yes	Yes	Federal government
**30. Antenatal corticosteroids given to women prior to birth to improve fetal, infant, child and adult health: New Zealand and Australian clinical practice guidelines**	https://ranzcog.edu.au/RANZCOG_SITE/media/RANZCOG-MEDIA/Women%27s%20Health/Statement%20and%20guidelines/Clinical-Obstetrics/Liggins-Institute-Antenatal-Corticosteroid-Clinical-Practice-Guidelines.pdf?ext=.pdf	2015	Anaesthetics	No	Yes	Other

Guideline name	URL	Year	Health topic	NHMRC-approved	GRADE	Funding source
**31. Acute pain management: scientific evidence fourth edition 2015**	https://onlinelibrary.wiley.com/doi/abs/10.5694/mja16.00133	2015	Anaesthetics	No	No	Other
**32. The 2020 Australian guideline for prevention, diagnosis and management of acute rheumatic fever and rheumatic heart disease**	https://onlinelibrary.wiley.com/doi/10.5694/mja2.50851	2020	Cardiology	No	Yes	Federal government
**33. Acute pain management: scientific evidence (5th edition)**	https://www.anzca.edu.au/getattachment/38ed54b7-fd19-4891-9ece-40d2f03b24f9/Acute-Pain-Management-Scientific-Evidence-5th-edition#page=	2020	Anaesthetics	No	No	Not stated
**34. ALS training**	https://regroup-production.s3.amazonaws.com/documents/ReviewReference/350660467/2020-ALS%20Training.pdf?response-content-type=application%2Fpdf&X-Amz-Algorithm=AWS4-HMAC-SHA256&X-Amz-Credential=AKIAYSFKCAWY23RWESRS%2F20220911%2Fus-east-1%2Fs3%2Faws4_request&X-Amz-Date=20220911T055749Z&X-Amz-Expires=604800&X-Amz-SignedHeaders=host&X-Amz-Signature=31c4aff5316d0d90880fdb8571db53fca915ba318c6e7d086bcbabd0b1d16430	2020	Emergency	No	No	Not stated
**35. Guidance for the management of early breast cancer: recommendations and practice points**	https://regroup-production.s3.amazonaws.com/documents/ReviewReference/350660448/2020-Guidance%20for%20the%20management%20of%20early%20brea%20%282%29.pdf?response-content-type=application%2Fpdf&X-Amz-Algorithm=AWS4-HMAC-SHA256&X-Amz-Credential=AKIAYSFKCAWY23RWESRS%2F20220911%2Fus-east-1%2Fs3%2Faws4_request&X-Amz-Date=20220911T055846Z&X-Amz-Expires=604800&X-Amz-SignedHeaders=host&X-Amz-Signature=b80f78430e6a0523f9935f270cde15519f2288cd2f39c7ad20699dfa002e2fdc	2020	Cancer	No	No	Not stated
**36. Diagnosis and management of melanoma**	https://wiki.cancer.org.au/Australia/Guidelines:Melanoma	2020	Cancer	No	Yes	Not stated
**37. Consensus statement on rural and remote cardiology during the covid-19 pandemic**	https://www.ncbi.nlm.nih.gov/pmc/articles/PMC7203036/	2020	Cardiology	No	Not	Not stated
**38. Evidence report for investigating symptoms of lung cancer: a guide for all health professionals**	https://regroup-production.s3.amazonaws.com/documents/ReviewReference/350660459/2020-Evidence%20Report%20for%20Investigating%20symptom.pdf?response-content-type=application%2Fpdf&X-Amz-Algorithm=AWS4-HMAC-SHA256&X-Amz-Credential=AKIAYSFKCAWY23RWESRS%2F20220911%2Fus-east-1%2Fs3%2Faws4_request&X-Amz-Date=20220911T060447Z&X-Amz-Expires=604800&X-Amz-SignedHeaders=host&X-Amz-Signature=ba773de7f3e38a032ffdf0718c64464257cd57cf68a8c40d99fdbd0a56356d1b	2020	Cancer	No	No	Not stated

Guideline name	URL	Year	Health topic	NHMRC-approved	GRADE	Funding source
**39. Consensus statement for patients with genetic heart disease and covid-19**	https://regroup-production.s3.amazonaws.com/documents/ReviewReference/350660396/2020-Consensus%20Statement%20for%20Patients%20with%20Gen.pdf?response-content-type=application%2Fpdf&X-Amz-Algorithm=AWS4-HMAC-SHA256&X-Amz-Credential=AKIAYSFKCAWY23RWESRS%2F20220911%2Fus-east-1%2Fs3%2Faws4_request&X-Amz-Date=20220911T060528Z&X-Amz-Expires=604800&X-Amz-SignedHeaders=host&X-Amz-Signature=1a82f35b709db4ca9e18d002f298ffcb06e44ac22b4ee112fef99d1a75c74251	2020	Cardiology	No	No	Not stated
**40. Position statement on the initial certification and maintenance of recognition in cardiac MRI**	https://regroup-production.s3.amazonaws.com/documents/ReviewReference/350660344/2020-Position%20Statement%20on%20the%20Initial%20Certifi.pdf?response-content-type=application%2Fpdf&X-Amz-Algorithm=AWS4-HMAC-SHA256&X-Amz-Credential=AKIAYSFKCAWY23RWESRS%2F20220911%2Fus-east-1%2Fs3%2Faws4_request&X-Amz-Date=20220911T060602Z&X-Amz-Expires=604800&X-Amz-SignedHeaders=host&X-Amz-Signature=4771d3bdc5fef31da0813d37edd8016824fec72f9151a0acfdb6f2209ff6c966	2020	Cardiology	No	No	Not stated
**41. Clinical practice guideline for care around stillbirth and neonatal death**	https://stillbirthcre.org.au/wp-content/uploads/2021/03/Clinical-Practice-Guidelines-for-Care-Around-Stillbirth-and-Neonatal-Death2-2.pdf	2020	Women's Health	No	No	Not stated
**42. Position statement on covid-19 and acute heart failure: screening the critically ill**	https://regroup-production.s3.amazonaws.com/documents/ReviewReference/350660397/2020-Position%20Statement%20on%20COVID-19%20and%20Acute.pdf?response-content-type=application%2Fpdf&X-Amz-Algorithm=AWS4-HMAC-SHA256&X-Amz-Credential=AKIAYSFKCAWY23RWESRS%2F20220911%2Fus-east-1%2Fs3%2Faws4_request&X-Amz-Date=20220911T061003Z&X-Amz-Expires=604800&X-Amz-SignedHeaders=host&X-Amz-Signature=b9bbb2cd27e75342ee4e82412ef5a0973f1b50509dd04bb94f107c7712ad4274	2020	Cardiology	No	No	Not stated
**43. Position statement on the evaluation of patients presenting with suspected acute coronary syndromes during the covid-19 pandemic**	https://regroup-production.s3.amazonaws.com/documents/ReviewReference/350660395/2020-Position%20Statement%20on%20the%20Evaluation%20of%20P.pdf?response-content-type=application%2Fpdf&X-Amz-Algorithm=AWS4-HMAC-SHA256&X-Amz-Credential=AKIAYSFKCAWY23RWESRS%2F20220911%2Fus-east-1%2Fs3%2Faws4_request&X-Amz-Date=20220911T061041Z&X-Amz-Expires=604800&X-Amz-SignedHeaders=host&X-Amz-Signature=fc2cc49c2f1d008e87d1d43fc1d281aa071fa96084a2958ac1798ce956c3ca8d	2020	Cardiology	No	No	Not stated

Guideline name	URL	Year	Health topic	NHMRC-approved	GRADE	Funding source
**44. Position statement on the management of cardiac electrophysiology and cardiac implantable electronic devices in Australia during the covid-19 pandemic: a living document version 2**	https://regroup-production.s3.amazonaws.com/documents/ReviewReference/350660393/2020-Position%20Statement%20on%20the%20Management%20of%20C.pdf?response-content-type=application%2Fpdf&X-Amz-Algorithm=AWS4-HMAC-SHA256&X-Amz-Credential=AKIAYSFKCAWY23RWESRS%2F20220911%2Fus-east-1%2Fs3%2Faws4_request&X-Amz-Date=20220911T061105Z&X-Amz-Expires=604800&X-Amz-SignedHeaders=host&X-Amz-Signature=9edba2186f4a23e08e119428397ccf354c1e08cb8e5c24f86cd6fe56ef569738	2020	Cardiology	No	No	Not stated
**45. Consensus guidelines for interventional cardiology services delivery during covid-19 pandemic in Australia and New Zealand**	https://linkinghub.elsevier.com/retrieve/pii/S1443950620301268	2020	Cardiology	No	No	Not stated
**46. Managing haematology and oncology patients during the covid-19 pandemic: interim consensus guidance**	https://onlinelibrary.wiley.com/doi/full/10.5694/mja2.50607	2020	Infectious Diseases	No	No	Not stated
**47. Australian and New Zealand consensus statement on the management of lymphoma, chronic lymphocytic leukaemia and myeloma during the covid-19 pandemic**	https://onlinelibrary.wiley.com/doi/10.1111/imj.14859	2020	Haematology	No	No	None
**48. The 2020 Royal Australian and New Zealand College of Psychiatrists clinical practice guidelines for mood disorders: major depression summary**	https://onlinelibrary.wiley.com/doi/10.1111/bdi.13035	2020	Psychiatry	No	Yes	Not stated
**49. The 2020 royal Australian and New Zealand college of psychiatrists clinical practice guidelines for mood disorders: bipolar disorder summary**	https://onlinelibrary.wiley.com/doi/10.1111/bdi.13036	2020	Psychiatry	No	Yes	Not stated
**50. Australian consensus statements for the regulation, production and use of faecal microbiota transplantation in clinical practice**	https://gut.bmj.com/content/69/5/801	2020	Gastroenterology	No	Yes	Other
**51. Nutrition management for critically and acutely unwell hospitalised patients with coronavirus disease 2019 (covid-19) in Australia and New Zealand**	https://www.Australiancriticalcare.com/article/S1036-7314(20)30244-7/fulltext	2020	Intensive care	No	No	Not stated
**52. Consensus statement: safe airway society principles of airway management and tracheal intubation specific to the covid-19 adult patient group**	https://onlinelibrary.wiley.com/doi/full/10.5694/mja2.50598	2020	Intensive care	No	No	Not stated

Guideline name	URL	Year	Health topic	NHMRC-approved	GRADE	Funding source
**53. Practical management of inflammatory bowel disease patients during the covid-19 pandemic: expert commentary from the gastroenterological society of Australia inflammatory bowel disease faculty**	https://onlinelibrary.wiley.com/doi/10.1111/imj.14889	2020	Gastroenterology	No	No	None
**54. Kidney biopsy**	https://regroup-production.s3.amazonaws.com/documents/ReviewReference/350660433/2019-Kidney%20Biopsy.pdf?response-content-type=application%2Fpdf&X-Amz-Algorithm=AWS4-HMAC-SHA256&X-Amz-Credential=AKIAYSFKCAWY23RWESRS%2F20220911%2Fus-east-1%2Fs3%2Faws4_request&X-Amz-Date=20220911T061800Z&X-Amz-Expires=604800&X-Amz-SignedHeaders=host&X-Amz-Signature=70785014a231e9bb3820ba2f459f148b4864ffeebe38454c44ff613b5d625ba7	2019	Nephrology	No	Yes	Other
**55. New guidelines from the thrombosis and haemostasis society of Australia and New Zealand for the diagnosis and management of venous thromboembolism**	https://onlinelibrary.wiley.com/doi/abs/10.5694/mja2.50004	2019	Haematology	No	Yes	Not stated
**56. Clinical practice guidelines for the prevention, early detection and management of colorectal cancer**	https://wiki.cancer.org.au/Australiawiki/images/e/ed/Colorectal_cancer_guidelines_short_form.pdf	2018	Cancer	No	Yes	Federal government
**57. Childhood hearing Australasian medical professionals network: consensus guidelines on investigation and clinical management of childhood hearing loss**	https://onlinelibrary.wiley.com/doi/10.1111/jpc.14508	2019	Paediatrics	No	Yes	Other
**58. Australian clinical consensus guideline: the diagnosis and acute management of childhood stroke**	https://journals.sagepub.com/doi/10.1177/1747493018799958	2019	Paediatrics	No	Yes	Other
**59. ANZSREI consensus statement on elective oocyte cryopreservation**	https://obgyn.onlinelibrary.wiley.com/doi/10.1111/ajo.13028	2019	Women's Health	No	Yes	None
**60. Diagnosis and management of heparin-induced thrombocytopenia: a consensus statement from the Thrombosis and Haemostasis Society of Australia and New Zealand hit writing group**	https://onlinelibrary.wiley.com/doi/abs/10.5694/mja2.50213	2019	Haematology	No	Yes	Not stated

Guideline name	URL	Year	Health topic	NHMRC-approved	GRADE	Funding source
**61. Clinical guidelines for stroke management 2019 update**	https://regroup-production.s3.amazonaws.com/documents/ReviewReference/350660282/2019-Clinical%20Guidelines%20for%20Stroke%20Management.pdf?response-content-type=application%2Fpdf&X-Amz-Algorithm=AWS4-HMAC-SHA256&X-Amz-Credential=AKIAYSFKCAWY23RWESRS%2F20220911%2Fus-east-1%2Fs3%2Faws4_request&X-Amz-Date=20220911T062623Z&X-Amz-Expires=604800&X-Amz-SignedHeaders=host&X-Amz-Signature=4265864575dd23c9b008b846562ae9fc7ab5d75d99b3e3ec59306646bc3598a3	2019	Neurology	Yes	Yes	Federal government
**62. Clinical guidelines for stroke management 2020 update**	https://informme.org.au/guidelines	2019	Neurology	Yes	Yes	Federal government
**63. The Australian immunisation handbook (2019)**	https://immunisationhandbook.health.gov.au/	2019	Immunology	Yes	Yes	Federal government
**64. Hepatitis b management during immunosuppression for haematological and solid organ malignancies: an Australian consensus statement**	https://onlinelibrary.wiley.com/doi/abs/10.5694/mja2.50160	2019	Infectious diseases	No	Yes	Other
**65. Development of standardised programme content for phase ii cardiac rehabilitation programmes in Australia using a modified Delphi process**	https://bmjopen.bmj.com/content/9/12/e032279	2019	Cardiology	No	No	Other
**66. COSA guidelines for the safe prescribing, dispensing and administration of systemic cancer therapy**	https://regroup-production.s3.amazonaws.com/documents/ReviewReference/350660190/2018-COSA%20guidelines%20for%20the%20safe%20prescribing.pdf?response-content-type=application%2Fpdf&X-Amz-Algorithm=AWS4-HMAC-SHA256&X-Amz-Credential=AKIAYSFKCAWY23RWESRS%2F20220911%2Fus-east-1%2Fs3%2Faws4_request&X-Amz-Date=20220911T063830Z&X-Amz-Expires=604800&X-Amz-SignedHeaders=host&X-Amz-Signature=8ce63bb27a2883d211b9f757808899dc6ab2abb174938ec9b7ba44b7cfd696db	2018	Cancer	No	Yes	Not stated
**67. Early detection of cancer in adolescent & young adults**	https://wiki.cancer.org.au/Australia/COSA:Early_detection_of_cancer_in_AYAs	2018	Cancer	No	Yes	Federal government
**68. National Heart Foundation of Australia and the Cardiac Society of Australia and New Zealand: Australian clinical guidelines for the diagnosis and management of atrial fibrillation 2018**	https://onlinelibrary.wiley.com/doi/abs/10.5694/mja18.00646	2018	Cardiology	No	Yes	Not stated
**69. Cancer pain management in adults**	https://wiki.cancer.org.au/Australia/Guidelines:Cancer_pain_management	2018	Cancer	No	No	Other

Guideline name	URL	Year	Health topic	NHMRC-approved	GRADE	Funding source
**70. Dialysis - infection control for haemodialysis units**	https://regroup-production.s3.amazonaws.com/documents/ReviewReference/350660272/2018-Dialysis%20-%20Infection%20Control%20for%20Haemodia.pdf?response-content-type=application%2Fpdf&X-Amz-Algorithm=AWS4-HMAC-SHA256&X-Amz-Credential=AKIAYSFKCAWY23RWESRS%2F20220911%2Fus-east-1%2Fs3%2Faws4_request&X-Amz-Date=20220911T064112Z&X-Amz-Expires=604800&X-Amz-SignedHeaders=host&X-Amz-Signature=903edfe9b2e09c45dda21180dd36df33b0e84b9d1b3e025e499ac263e9ee3664	2018	Nephrology	No	Yes	Not stated
**71. Guidance on the clinical management of anxiety disorders, specifically focusing on diagnosis and treatment strategies**	https://regroup-production.s3.amazonaws.com/documents/ReviewReference/350660280/2018-Guidance%20on%20the%20clinical%20management%20of%20an.pdf?response-content-type=application%2Fpdf&X-Amz-Algorithm=AWS4-HMAC-SHA256&X-Amz-Credential=AKIAYSFKCAWY23RWESRS%2F20220911%2Fus-east-1%2Fs3%2Faws4_request&X-Amz-Date=20220911T064138Z&X-Amz-Expires=604800&X-Amz-SignedHeaders=host&X-Amz-Signature=4ecb1415bc9c11c8e68c09205f510adb87225d2c2a339c9d0574e1eef0e534f3	2018	Psychiatry	No	No	Other
**72. National Heart Foundation of Australia and Cardiac Society of Australia and New Zealand: guidelines for the prevention, detection, and management of heart failure in Australia 2018**	https://regroup-production.s3.amazonaws.com/documents/ReviewReference/350660288/2018-National%20Heart%20Foundation%20of%20Australia%20an%20%282%29.pdf?response-content-type=application%2Fpdf&X-Amz-Algorithm=AWS4-HMAC-SHA256&X-Amz-Credential=AKIAYSFKCAWY23RWESRS%2F20220911%2Fus-east-1%2Fs3%2Faws4_request&X-Amz-Date=20220911T064327Z&X-Amz-Expires=604800&X-Amz-SignedHeaders=host&X-Amz-Signature=d4b209310d4711d9cf8d30b1351129538c2f4043f7fd299a12c85c8ac51810ec	2018	Cardiology	No	Yes	Not stated
**73. Methods of melanoma detection and of skin monitoring for individuals at high risk of melanoma: new Australian clinical practice guidelines**	https://onlinelibrary.wiley.com/doi/abs/10.5694/mja2.12033	2019	Cancer	No	Yes	Other
**74. Putting prevention into practice - guidelines for the implementation of prevention in the general practice setting**	https://regroup-production.s3.amazonaws.com/documents/ReviewReference/350660060/2018-Putting%20prevention%20into%20practice.pdf?response-content-type=application%2Fpdf&X-Amz-Algorithm=AWS4-HMAC-SHA256&X-Amz-Credential=AKIAYSFKCAWY23RWESRS%2F20220911%2Fus-east-1%2Fs3%2Faws4_request&X-Amz-Date=20220911T131023Z&X-Amz-Expires=604800&X-Amz-SignedHeaders=host&X-Amz-Signature=567b3224aefca1c548fcfbf61145a0248ba42b45c94c12fd68581f675b5ac8d5	2018	General Practice	No	No	Other

Guideline name	URL	Year	Health topic	NHMRC-approved	GRADE	Funding source
**75. Royal Australian and New Zealand College of Psychiatrists clinical practice guidelines for mood disorders: major depression summary**	https://onlinelibrary.wiley.com/doi/abs/10.5694/mja17.00659	2018	Psychiatry	No	No	Other
**76. Royal Australian and New Zealand College of Psychiatrists clinical practice guidelines for mood disorders: bipolar disorder summary**	https://onlinelibrary.wiley.com/doi/abs/10.5694/mja17.00658	2018	Psychiatry	No	No	Other
**77. The lung cancer framework: principles for best practice lung cancer care in Australia**	https://regroup-production.s3.amazonaws.com/documents/ReviewReference/350660197/2018-The%20Lung%20Cancer%20Framework_%20Principles%20for.pdf?response-content-type=application%2Fpdf&X-Amz-Algorithm=AWS4-HMAC-SHA256&X-Amz-Credential=AKIAYSFKCAWY23RWESRS%2F20220911%2Fus-east-1%2Fs3%2Faws4_request&X-Amz-Date=20220911T131321Z&X-Amz-Expires=604800&X-Amz-SignedHeaders=host&X-Amz-Signature=4c5bd42a0275886a94aeff80f79fe8e746f3c88fd0ca3600dd99150d6b6e313a	2018	Cancer	No	No	Federal government
**78. Updated clinical practice guidelines on pregnancy care**	https://onlinelibrary.wiley.com/doi/abs/10.5694/mja18.00286	2018	Women's Health	Yes	Yes	Federal government
**79. Diagnosis and management of idiopathic pulmonary fibrosis: Thoracic Society of Australia and New Zealand and Lung Foundation Australia position statements summary**	https://regroup-production.s3.amazonaws.com/documents/ReviewReference/351122822/Australia-2012-Australian%20guideline%20for%20preven.pdf?response-content-type=application%2Fpdf&X-Amz-Algorithm=AWS4-HMAC-SHA256&X-Amz-Credential=AKIAYSFKCAWY23RWESRS%2F20220911%2Fus-east-1%2Fs3%2Faws4_request&X-Amz-Date=20220911T131432Z&X-Amz-Expires=604800&X-Amz-SignedHeaders=host&X-Amz-Signature=a0143b91fa15479df8cb9acb11365246024f1681a826f24262eeb8fff2bfcb20	2018	Respiratory	No	No	Not stated
**80. Revised Australian national guidelines for colorectal cancer screening: family history**	https://onlinelibrary.wiley.com/doi/abs/10.5694/mja18.00142	2018	Cancer	Yes	No	Not stated
**81. Clinical practice guidelines for the treatment of lung cancer**	https://wiki.cancer.org.au/Australiawiki/index.php?title=Guidelines:Lung_cancer/Guideline_development_process&oldid=47792	2018	Cancer	No	No	Federal government
**82. Clinical Oncology Society of Australia position statement on exercise in cancer care**	https://onlinelibrary.wiley.com/doi/abs/10.5694/mja18.00199	2018	Cancer	No	No	Note stated
**83. Management of menopausal symptoms in women with a history of breast cancer**	https://www.cancerAustralia.gov.au/resources/clinical-practice-guidelines/menopausal-guidelines	2017	Cancer	No	No	Federal government
**84. The 2016 royal Australian and New Zealand College of Psychiatrists guidelines for the management of schizophrenia and related disorders**	https://onlinelibrary.wiley.com/doi/abs/10.5694/mja16.01159	2016	Psychiatry	No	Not	Not stated

Guideline name	URL	Year	Health topic	NHMRC-approved	GRADE	Funding source
**85. The Australasian Society for Infectious Diseases and Refugee Health Network of Australia recommendations for health assessment for people from refugee-like backgrounds: an abridged outline**	https://onlinelibrary.wiley.com/doi/abs/10.5694/mja16.00826	2017	Infectious diseases	No	No	Not stated
**86. The diagnosis and acute management of childhood stroke, clinical guideline**	https://regroup-production.s3.amazonaws.com/documents/ReviewReference/350659900/2017-The%20Diagnosis%20and%20Acute%20Management%20of%20Chi.pdf?response-content-type=application%2Fpdf&X-Amz-Algorithm=AWS4-HMAC-SHA256&X-Amz-Credential=AKIAYSFKCAWY23RWESRS%2F20220911%2Fus-east-1%2Fs3%2Faws4_request&X-Amz-Date=20220911T132058Z&X-Amz-Expires=604800&X-Amz-SignedHeaders=host&X-Amz-Signature=0afa2a87701f5c21edfcbe502b89c30f455b19eb52d95bec0dcea853ae87b717	2017	Neurology	No	Yes	Other
**87. An Australian consensus on infant feeding guidelines to prevent food allergy: outcomes from the Australian infant feeding summit**	https://www.jaci-inpractice.org/article/S2213-2198(17)30184-8/fulltext	2017	Immunology	No	No	NHMRC
**88. Treatment of patients with Waldenstram macroglobulinaemia: clinical practice guidelines from the Myeloma Foundation of Australia medical and scientific advisory group**	https://onlinelibrary.wiley.com/doi/10.1111/imj.13311	2017	Cancer	No	No	None
**89. A collaborative approach to adopting/adapting guidelines - the Australian 24-hour movement guidelines for the early years (birth to 5 years): an integration of physical activity, sedentary behavior, and sleep**	https://bmcpublichealth.biomedcentral.com/articles/10.1186/s12889-017-4867-6	2017	Exercise medicine	No	No	Federal government
**90. Royal Australian and New Zealand College of Psychiatrists expert consensus statement for the treatment, management and monitoring of the physical health of people with an enduring psychotic illness**	https://journals.sagepub.com/doi/10.1177/0004867416686693	2017	Infectious diseases	No	No	None
**91. Endocrine Society of Australia position statement on male hypogonadism (part 1): assessment and indications for testosterone therapy**	https://onlinelibrary.wiley.com/doi/abs/10.5694/mja16.00393	2016	Endocrinology	No	No	Not stated
**92. Endocrine Society of Australia position statement on male hypogonadism (part 2): treatment and therapeutic considerations**	https://onlinelibrary.wiley.com/doi/abs/10.5694/mja16.00448	2016	Endocrinology	No	No	Not stated

Guideline name	URL	Year	Health topic	NHMRC-approved	GRADE	Funding source
**93. General practice management of type 2 diabetes 2016**	https://regroup-production.s3.amazonaws.com/documents/ReviewReference/350659655/Generalpracticemanagementoftype2diabetes201618.pdf?response-content-type=application%2Fpdf&X-Amz-Algorithm=AWS4-HMAC-SHA256&X-Amz-Credential=AKIAYSFKCAWY23RWESRS%2F20220911%2Fus-east-1%2Fs3%2Faws4_request&X-Amz-Date=20220911T132819Z&X-Amz-Expires=604800&X-Amz-SignedHeaders=host&X-Amz-Signature=58a6d0478a1aefc4d08b48122c0693f0ad4679525577a8108981c8040cf7a514	2016	Endocrinology	No	No	Not stated
**94. Guidance addressing all aspects of the care of people with schizophrenia and related disorders. Includes correct diagnosis, symptom relief and recovery of social function**	https://regroup-production.s3.amazonaws.com/documents/ReviewReference/350660045/2016-Guidance%20addressing%20all%20aspects%20of%20the%20ca.pdf?response-content-type=application%2Fpdf&X-Amz-Algorithm=AWS4-HMAC-SHA256&X-Amz-Credential=AKIAYSFKCAWY23RWESRS%2F20220911%2Fus-east-1%2Fs3%2Faws4_request&X-Amz-Date=20220911T132853Z&X-Amz-Expires=604800&X-Amz-SignedHeaders=host&X-Amz-Signature=0cc6f305c49a8fd965f9d5a6e0ee814d1125ab306360fb6d25ef16e51bd211bd	2016	Psychiatry	No	No	Other
**95. Guidance on the clinical management of deliberate self-harm (dsh) and on the organisation and delivery of services**	https://regroup-production.s3.amazonaws.com/documents/ReviewReference/350660044/2016-Guidance%20on%20the%20clinical%20management%20of%20de.pdf?response-content-type=application%2Fpdf&X-Amz-Algorithm=AWS4-HMAC-SHA256&X-Amz-Credential=AKIAYSFKCAWY23RWESRS%2F20220911%2Fus-east-1%2Fs3%2Faws4_request&X-Amz-Date=20220911T132916Z&X-Amz-Expires=604800&X-Amz-SignedHeaders=host&X-Amz-Signature=80cfcdf720fb179f0c1cacb5c5c2d44d2f88aa4a4c581be5bd6a8e58ba68fe72	2016	Psychiatry	No	No	Other
**96. Guideline for the diagnosis and management of hypertension in adults 2016**	https://onlinelibrary.wiley.com/doi/abs/10.5694/mja16.00526	2016	Cardiology	No	Yes	Not stated
**97. Guidelines for the management of screen-detected abnormalities, screening in specific populations and investigation of abnormal vaginal bleeding**	https://regroup-production.s3.amazonaws.com/documents/ReviewReference/350660215/2016-Guidelines%20for%20the%20management%20of%20screen-d.pdf?response-content-type=application%2Fpdf&X-Amz-Algorithm=AWS4-HMAC-SHA256&X-Amz-Credential=AKIAYSFKCAWY23RWESRS%2F20220911%2Fus-east-1%2Fs3%2Faws4_request&X-Amz-Date=20220911T132958Z&X-Amz-Expires=604800&X-Amz-SignedHeaders=host&X-Amz-Signature=b7e17efd56595e61beba4f0e9c25ed2f7c83cbee4a3e8e37daf95a632308e8ed	2016	Cancer	No	No	Federal government

Guideline name	URL	Year	Health topic	NHMRC-approved	GRADE	Funding source
**98. Australasian Society of Infectious Diseases updated guidelines for the management of clostridium difficile infection in adults and children in Australia and New Zealand**	https://onlinelibrary.wiley.com/doi/10.1111/imj.13027	2016	Infectious diseases	No	No	None
**99. Optimizing combination dabrafenib and trametinib therapy in braf mutation-positive advanced melanoma patients: guidelines from Australian melanoma medical oncologists**	https://onlinelibrary.wiley.com/doi/10.1111/ajco.12656	2016	Cancer	No	No	Other
**100. National Heart Foundation of Australia & Cardiac Society of Australia and New Zealand: Australian clinical guidelines for the management of acute coronary syndromes 2016**	https://onlinelibrary.wiley.com/doi/abs/10.5694/mja16.00368	2016	Cardiology	No	Yes	Not stated
**101. Clinical practice guidelines for the treatment and management of endometrial cancer**	https://wiki.cancer.org.au/Australia/Guidelines:Endometrial_cancer/Treatment/Early_stage	2016	Cancer	No	No	Federal government
**102. Physiotherapy for cystic fibrosis in Australia and New Zealand: a clinical practice guideline**	https://onlinelibrary.wiley.com/doi/10.1111/resp.12764	2016	Orthopaedics	No	No	Other
**103. Guidelines for timely initiation of chemotherapy: a proposed framework for access to medical oncology and haematology cancer clinics and chemotherapy services**	https://onlinelibrary.wiley.com/doi/10.1111/imj.13157	2016	Cancer	No	No	Other
**104. Guidance on the clinical management of depressive and bipolar disorders, specifically focusing on diagnosis and treatment strategies**	https://www.ranzcp.org/files/resources/college_statements/clinician/cpg/mood-disorders-cpg.aspx	2015	Psychiatry	No	No	Other
**105. Chronic suppurative lung disease and bronchiectasis in children and adults in Australia and New Zealand Thoracic Society of Australia and New Zealand guidelines**	https://onlinelibrary.wiley.com/doi/abs/10.5694/mja14.00287	2014	Respiratory	No	Yes	NHMRC
**106. KHA-CARI guideline recommendations for the diagnosis and management of autosomal dominant polycystic kidney disease**	https://regroup-production.s3.amazonaws.com/documents/ReviewReference/350659656/2015-Autosomal%20Dominant%20Polycystic%20Kidney%20Dise.pdf?response-content-type=application%2Fpdf&X-Amz-Algorithm=AWS4-HMAC-SHA256&X-Amz-Credential=AKIAYSFKCAWY23RWESRS%2F20220911%2Fus-east-1%2Fs3%2Faws4_request&X-Amz-Date=20220911T133820Z&X-Amz-Expires=604800&X-Amz-SignedHeaders=host&X-Amz-Signature=155b5d816456e499c2c46080b7c93b83496f9e080e8705d39862130cf1523516	2015	Nephrology	No	No	Not stated

Guideline name	URL	Year	Health topic	NHMRC-approved	GRADE	Funding source
**107. PSA testing and early management of test-detected prostate cancer**	https://wiki.cancer.org.au/australiawiki/images/1/1b/PSA_Testing_and_Early_Management_of_Test-detected_Prostate_Cancer_-_Clinical_practice_guidelines_-_Nov15.pdf	2015	Cancer	Yes	No	Not stated
**108. Australian hip surveillance guidelines for children with cerebral palsy: 5-year review**	https://onlinelibrary.wiley.com/doi/10.1111/dmcn.12754	2015	Paediatrics	No	No	Not stated
**109. Consensus guidelines for the investigation and management of encephalitis in adults and children in Australia and New Zealand**	https://onlinelibrary.wiley.com/doi/10.1111/imj.12749	2015	Infectious diseases	No	No	Not stated
**110. Treatment of patients with multiple myeloma who are eligible for stem cell transplantation: position statement of the myeloma foundation of Australia medical and scientific advisory group**	https://onlinelibrary.wiley.com/doi/10.1111/imj.12640	2015	Cancer	No	No	None
**111. Guidelines for safe practice of stereotactic body (ablative) radiation therapy**	https://onlinelibrary.wiley.com/doi/10.1111/1754-9485.12336	2015	Cancer	No	No	Not stated
**112. Australian STI management guidelines for use in primary care**	https://sti.guidelines.org.au/committees/	2017	Sexual health	No	No	Federal government
**113. Clinical guidance for responding to suffering in adults with cancer**	https://cancerwa.asn.au/resources/2020-12-02-clinical-guidance-adults-with-cancer.pdf	2014	Cancer	No	No	Federal government
**114. Recommendations for the management of central nervous system (CNS) metastases in women with secondary breast cancer**	https://www.canceraustralia.gov.au/system/tdf/guidelines/cns_metastases_in_women_with_secondary_breast_cancer.pdf?file=1&type=node&id=3752	2014	Cancer	No	No	Federal government
**115. Supporting smoking cessation: a guide for health professionals**	https://regroup-production.s3.amazonaws.com/documents/ReviewReference/350659418/2014-Supporting%20smoking%20cessation_%20A%20guide%20for.pdf?response-content-type=application%2Fpdf&X-Amz-Algorithm=AWS4-HMAC-SHA256&X-Amz-Credential=AKIAYSFKCAWY23RWESRS%2F20220911%2Fus-east-1%2Fs3%2Faws4_request&X-Amz-Date=20220911T135322Z&X-Amz-Expires=604800&X-Amz-SignedHeaders=host&X-Amz-Signature=66bcadb0bb8b7005f5f98434442acf654e531b93b6d81f5a6f482edc697b48f0	2014	General practice	No	Yes	Federal government
**116. Treatment and prevention of mycobacterium ulcerans infection (Buruli ulcer) in Australia: guideline update**	https://onlinelibrary.wiley.com/doi/10.5694/mja13.11331	2014	Infectious diseases	No	No	other

Guideline name	URL	Year	Health topic	NHMRC-approved	GRADE	Funding source
**117. Abuse and violence - working with our patients in general practice**	https://regroup-production.s3.amazonaws.com/documents/ReviewReference/350659477/2014-Abuse%20and%20violence%20-%20working%20with%20our%20pat.pdf?response-content-type=application%2Fpdf&X-Amz-Algorithm=AWS4-HMAC-SHA256&X-Amz-Credential=AKIAYSFKCAWY23RWESRS%2F20220911%2Fus-east-1%2Fs3%2Faws4_request&X-Amz-Date=20220911T135641Z&X-Amz-Expires=604800&X-Amz-SignedHeaders=host&X-Amz-Signature=e5c4fca6e64a2ae799fef903341334f92bf481ddc1a89a7ee536708a79a57dcc	2014	General practice	No	No	Federal government
**118. A systematic approach to chronic heart failure care: a consensus statement**	https://onlinelibrary.wiley.com/doi/abs/10.5694/mja14.00032	2014	General practice	No	Yes	None
**119. Australian consensus guidelines for the safe handling of monoclonal antibodies for cancer treatment by healthcare personnel**	https://onlinelibrary.wiley.com/doi/10.1111/imj.12564	2014	Cancer	No	No	Other
**120. Australian guidelines for the treatment of acute stress disorder and posttraumatic stress disorder**	https://phoenixaustralia.org/wp-content/uploads/2015/03/Phoenix-ASD-PTSD-Guidelines.pdf	2013	Psychiatry	Yes	No	Federal government
**121. Clinical practice guidelines for the management of rotator cuff syndrome in the workplace**	https://regroup-production.s3.amazonaws.com/documents/ReviewReference/350659427/2013-Clinical%20practice%20guidelines%20for%20the%20mana.pdf?response-content-type=application%2Fpdf&X-Amz-Algorithm=AWS4-HMAC-SHA256&X-Amz-Credential=AKIAYSFKCAWY23RWESRS%2F20220911%2Fus-east-1%2Fs3%2Faws4_request&X-Amz-Date=20220911T135826Z&X-Amz-Expires=604800&X-Amz-SignedHeaders=host&X-Amz-Signature=91b64bcb9d67fc338c07af6e7a049292876bf4a78ed2eced37ba9d61858691ef	2013	Orthopaedics	No	No	Other
**122. Dialysis - biochemical and haematological targets**	https://www.cariguidelines.org/guidelines/dialysis/biochemical-and-haematological-targets/	2013	Nephrology	No	Yes	Other
**123. Psychosocial risk factors for coronary heart disease**	https://onlinelibrary.wiley.com/doi/10.5694/mja13.10440	2013	Cardiology	No	No	Other
**124. Building healthy bones throughout life. An evidence-informed strategy to prevent osteoporosis in Australia**	https://regroup-production.s3.amazonaws.com/documents/ReviewReference/350659432/Peter-2013-Building%20healthy%20bones%20throughout%20l.pdf?response-content-type=application%2Fpdf&X-Amz-Algorithm=AWS4-HMAC-SHA256&X-Amz-Credential=AKIAYSFKCAWY23RWESRS%2F20220911%2Fus-east-1%2Fs3%2Faws4_request&X-Amz-Date=20220911T140014Z&X-Amz-Expires=604800&X-Amz-SignedHeaders=host&X-Amz-Signature=80cbad1be51b70f4a88b56d8f54c22213f0591cbb3884d1e70684037ed10a06a	2013	Endocrinology	No	No	Federal government

Guideline name	URL	Year	Health topic	NHMRC-approved	GRADE	Funding source
**125. Australian dietary guidelines**	https://regroup-production.s3.amazonaws.com/documents/ReviewReference/351122828/National-2013-Australian%20Dietary%20Guidelines.pdf?response-content-type=application%2Fpdf&X-Amz-Algorithm=AWS4-HMAC-SHA256&X-Amz-Credential=AKIAYSFKCAWY23RWESRS%2F20220911%2Fus-east-1%2Fs3%2Faws4_request&X-Amz-Date=20220911T140041Z&X-Amz-Expires=604800&X-Amz-SignedHeaders=host&X-Amz-Signature=56f236f44eb59000a6997ab0f05177e5d383899ad36a1278e8ce26a2a5f10ec5	2013	Nutrition	No	No	Other
**126. Detection and management of mood disorders in the maternity setting: the Australian clinical practice guidelines**	https://www.sciencedirect.com/science/article/abs/pii/S1871519211002514?via%3Dihub	2012	Psychiatry	No	No	Other
**127. Chronic kidney disease and automatic reporting of estimated glomerular filtration rate - new developments and revised recommendations**	https://www.mja.com.au/system/files/issues/197_04_200812/joh11329_web_k_fm.pdf	2012	Nephrology	No	No	Other
**128. The Australian guideline for prevention, diagnosis and management of acute rheumatic fever and rheumatic heart disease in Australia (2nd edition)**	https://regroup-production.s3.amazonaws.com/documents/ReviewReference/350659402/2012-Rheumatic%20Fever%20and%20Rheumatic%20Heart%20Disea.pdf?response-content-type=application%2Fpdf&X-Amz-Algorithm=AWS4-HMAC-SHA256&X-Amz-Credential=AKIAYSFKCAWY23RWESRS%2F20220911%2Fus-east-1%2Fs3%2Faws4_request&X-Amz-Date=20220911T140233Z&X-Amz-Expires=604800&X-Amz-SignedHeaders=host&X-Amz-Signature=f6037aa5509c2405449a0717ae885abc7644c9f9c0220927f4479231e754384e	2012	Cardiology	No	No	Not stated
**129. Chronic kidney disease and measurement of albuminuria or proteinuria: a position statement**	https://onlinelibrary.wiley.com/doi/10.5694/mja11.11468	2012	Nephrology	No	No	Not stated
**130. Prevention of venous thromboembolism in patients admitted to Australian hospitals: summary of national health and medical research council clinical practice guideline**	https://onlinelibrary.wiley.com/doi/10.1111/j.1445-5994.2012.02808.x	2012	Haematology	Yes	No	NHMRC
**131. Care of kidney transplant recipients**	https://regroup-production.s3.amazonaws.com/documents/ReviewReference/350659413/2012-Care%20of%20Kidney%20Transplant%20Recipients.pdf?response-content-type=application%2Fpdf&X-Amz-Algorithm=AWS4-HMAC-SHA256&X-Amz-Credential=AKIAYSFKCAWY23RWESRS%2F20220911%2Fus-east-1%2Fs3%2Faws4_request&X-Amz-Date=20220911T140431Z&X-Amz-Expires=604800&X-Amz-SignedHeaders=host&X-Amz-Signature=11837a800872b90ad630079a16509a6dea6ccc4af675da211fb06156bf734532	2012	Nephrology	No	No	Other

Guideline name	URL	Year	Health topic	NHMRC-approved	GRADE	Funding source
**132. Clinical practice guideline: depression and related disorders - anxiety, bipolar disorder and puerperal psychosis - in the perinatal period**	https://regroup-production.s3.amazonaws.com/documents/ReviewReference/350659387/2011-Clinical%20Practice%20Guideline_%20Depression%20a.pdf?response-content-type=application%2Fpdf&X-Amz-Algorithm=AWS4-HMAC-SHA256&X-Amz-Credential=AKIAYSFKCAWY23RWESRS%2F20220911%2Fus-east-1%2Fs3%2Faws4_request&X-Amz-Date=20220911T140508Z&X-Amz-Expires=604800&X-Amz-SignedHeaders=host&X-Amz-Signature=f3c7a56b97942f89a1126e48469214eb3026abafc44d25e96c648fa86ceaf510	2011	Psychiatry	Yes	No	Federal government
**133. Clinical practice guidelines: depression in adolescents and young adults**	http://www.24hmb.com/voimages/web_image//upload/file/20140614/20851402752241427.pdf	2011	Psychiatry	Yes	No	Other
**134. Recommendations for use of hypofractionated radiotherapy for early (operable) breast cancer**	https://www.cancerAustralia.gov.au/sites/default/files/publications/hyrad-recommendations-for-use-of-hypofractionated-radiotherapy-for-early-operable-breast-cancer_504af0326254b.pdf	2011	Cancer	No	No	Other
**135. Ambulatory blood pressure monitoring in Australia**	https://oce.ovid.com/article/00004872-201202000-00002/HTML	2011	Cardiology	No	No	Other
**136. 2011 addendum to the National Heart Foundation of Australia/Cardiac Society of Australia and New Zealand guidelines for the management of acute coronary syndromes (ACS) 2006**	https://www.csanz.edu.au/documents/guidelines/clinical_practice/2011_HF_CSANZ_ACS_Addendum_with_algorithm.pdf	2011	Cardiology	No	No	Not stated
**137. Cicada: cough in children and adults: diagnosis and assessment. Australian cough guidelines summary statement**	https://onlinelibrary.wiley.com/doi/abs/10.5694/j.1326-5377.2010.tb03504.x	20101	Respiratory	No	Yes	Not stated
**138. Recommendations for follow-up of women with early breast cancer**	https://www.cancerAustralia.gov.au/sites/default/files/publications/fueg-follow-up-of-women-with-early-breast-cancer_504af0340ef02.pdf	2010	Cancer	No	No	Not stated
**139. Chronic suppurative lung disease and bronchiectasis in children and adults in Australia and New Zealand. A position statement from the Thoracic Society of Australia and New Zealand and the Australian lung foundation**	https://onlinelibrary.wiley.com/doi/abs/10.5694/j.1326-5377.2010.tb03949.x	2010	Respiratory	No	Yes	NHMRC
**140. An Australian guideline on the diagnosis of overseas acquired Lyme disease/borreliosis**	https://www1.health.gov.au/internet/main/publishing.nsf/Content/cda-cdi3904-pdf-cnt.htm/$FILE/cdi3904d.pdf	2015	Infectious disease	No	No	Not stated
**141. ADIPS consensus guidelines for the testing and diagnosis of hyperglycaemia in pregnancy in Australia and New Zealand**	https://www.adips.org/downloads/2014ADIPSGDMGuidelinesV18.11.2014_000.pdf	2014	Endocrinology	No	No	Not stated
**142. ANZICS statement on care and decision-making at the end of life for the critically ill**	https://www.anzics.com.au/wp-content/uploads/2018/08/ANZICS-Statement-on-Care-and-Decision-Making-at-the-End-of-Life-for-the-Critically-Ill.pdf	2014	Intensive care	No	No	Not stated
**143. Australian and New Zealand guideline for hip fracture care - improving outcomes in hip fracture management of adults**	https://anzhfr.org/wp-content/uploads/2016/07/ANZ-Guideline-for-Hip-Fracture-Care.pdf	2014	Orthopaedics	Yes	Yes	Other

Guideline name	URL	Year	Health topic	NHMRC-approved	GRADE	Funding source
**144. Bone marrow specimen (aspirate and trephine biopsy) structured reporting protocol**	https://www.rcpa.edu.au/getattachment/a133bb88-cb34-47c6-9bb2-a66298595464/Protocol-bone-marrow-specimen.aspx	2014	Pathology	No	No	Not stated
**145. Cancer of the exocrine pancreas, ampulla of Vater and distal common bile duct structured reporting protocol (1st edition 2014)**	https://studylib.net/doc/12254616/cancer-of-the-exocrine-pancreas--ampulla-of-vater-and-distal	2014	Pathology	No	No	Not stated
**146. General practice management of type 2 diabetes 2014-15**	https://www.racgp.org.au/download/Documents/Guidelines/Diabetes/2014diabetesmanagement.pdf	2014	Endocrinology	No	No	Other
**147. Therapeutic guidelines endocrinology version 5,2014**	https://www.tg.org.au/the-organisation/expert-groups-2/endocrinology/	2014	Endocrinology	No	No	None
**148. Clinical practice guidelines for the management of Barrett's oesophagus and early oesophageal adenocarcinoma**	https://onlinelibrary.wiley.com/doi/10.1111/jgh.12913	2014	Cancer	No	No	Other
**149. Primary cutaneous melanoma structured reporting protocol. 2nd edition 2014**	https://www.rcpa.edu.au/getattachment/9f61b036-a56c-4f42-bf89-bffed98b5494/Protocol-primary-cutaneous-melanoma.aspx	2014	Pathology	No	No	Not stated
**150. Clinical practice guideline for acute kidney injury**	https://onlinelibrary.wiley.com/doi/pdfdirect/10.1111/nep.12220	2014	Nephrology	No	No	Not stated
**151. Recommendations for the identification and management of fear of cancer recurrence in adult cancer survivors**	https://www.canceraustralia.gov.au/guidelines/guideline_21.pdf	2014	Cancer	No	No	Not stated
**152. RANZP clinical practice guideline for the treatment of eating disorders**	https://www.ranzcp.org/files/resources/college_statements/clinician/cpg/eating-disorders-cpg.aspx	2014	Psychiatry	No	No	Other
**153. Clinical practice guidelines antenatal care - module 2**	https://consultations.health.gov.au/phd-tobacco/clinical-practice-guidelines-antenatal-care-module/supporting_documents/Antenatal%20module%20II%20diabetes%20consultation%2020Jan2014%20D14142528.PDF	2014	Women's health	No	No	Federal government
**154. Therapeutic guidelines antibiotic version 15**	https://www.tg.org.au/the-organisation/expert-groups-2/antibiotic/	2014	Infectious diseases	No	No	Not stated
**155. Trace element supplementation for parenteral nutrition guidelines**	http://auspen.org.au/?wpfb_dl=78&token=Hyypaom4jKiqWZSM7C6KZvRy9F5jnfeWQFf1iTV4cbfbLfFq3Gzmo1uSDfmY7hcR&TOPIC_ID=111340	2014	Nutrition	No	No	Not stated
**156. First line chemotherapy for the treatment of women with epithelial ovarian cancer**	https://www.canceraustralia.gov.au/system/tdf/guidelines/first-line_chemotherapy_for_the_treatment_of_women_with_epithelial_ovarian_cancer.pdf?file=1&type=node&id=3958	2014	Cancer	No	No	Not stated
**157. National guidelines for medication-assisted treatment of opioid dependence**	https://www.health.gov.au/sites/default/files/national-guidelines-for-medication-assisted-treatment-of-opioid-dependence.pdf	2014	Gambling and addiction	No	No	Other
**158. The COPDX plan - Australian and New Zealand guidelines for the management of chronic obstructive pulmonary disease 2014**	https://copdx.org.au/wp-content/uploads/2015/08/LFA-COPD-X-doc_V3.02_0815_WEB.pdf	2014	Respiratory	No	No	Other
**159. Intrapartum fetal surveillance clinical guideline**	https://fsep.ranzcog.edu.au/FSEP/media/FSEP/Documents/RANZCOG%20IFS%20Clinical%20Guideline%202014.pdf	2014	Women's health	No	No	Other

Guideline name	URL	Year	Health topic	NHMRC-approved	GRADE	Funding source
**160. Endoscopic resection (ER) of the oesophagus and gastro-oesophageal junction**	https://www.rcpa.edu.au/getattachment/ad73fab3-1e84-483f-a8a7-6c5281f04aa2/Protocol-endoscopic-resection-oesophagus.aspx	2013	Pathology	No	No	Not stated
**161. Vulva cancer structured reporting protocol**	https://www.rcpa.edu.au/getattachment/9cdcbca0-6523-4a13-b716-370d5bc945c3/Protocol-vulva-cancer.aspx	2013	Pathology	No	No	Not stated
**162. Clinical practice guidelines for the management of adult onset sarcoma**	https://wiki.cancer.org.au/australia/Guidelines:Sarcoma	2013	Cancer	No	No	Not stated
**163. The Australian immunisation handbook 10th edition**	https://doh.getquickmail.com/stockpdfs/1108-doh-immunisation-handbook-10th.pdf	2013	Immunology	Yes	No	Federal government
**164. Creutzfeldt-Jakob disease**	https://www1.health.gov.au/internet/main/publishing.nsf/content/3A968399995CFCE5CA257BF000211E32/$File/CJDInfectionControlGuidelinesJan2013.pdf	2013	Infectious diseases	No	No	Not stated
**165. Aboriginal and Torres strait islander health workers and blood-borne viruses**	https://www.ashm.org.au/resources/Aboriginal_and_Torres_Strait_Islander_Health_Workers_and_BBVs.pdf	2013	Infectious disease	No	No	Federal government
**166. Therapeutic guidelines psychotropic version 7**	https://www.tg.org.au/the-organisation/expert-groups/psychotropic/	2013	Psychiatry	No	No	Not stated
**167. Screening, referral and treatment for depression in patients with coronary heart disease**	https://www.mja.com.au/system/files/issues/198_09_200513/col10153_004_web_rtf_fm.pdf	2013	Cardiology	No	No	None
**168. Lung cancer structured reporting protocol. 2nd edition 2013**	https://www.rcpa.edu.au/getattachment/69ffe543-efb7-4de4-b18b-cee5c352a364/Protocol-lung-cancer.aspx	2013	Pathology	No	No	Not stated
**169. Clinical practice guidelines for the management of overweight and obesity in adults, adolescents and children in Australia**	https://www.nhmrc.gov.au/about-us/publications/clinical-practice-guidelines-management-overweight-and-obesity#block-views-block-file-attachments-content-block-1	2013	Public health	Yes	No	Federal government
**170. Cervical cancer structured reporting protocol (1st edition 2013)**	https://www.rcpa.edu.au/getattachment/2dfcc534-547d-455a-837b-79bfeb2b60e7/Protocol-Cervical-cancer.aspx	2013	Pathology	No	No	Not stated
**171. Recommendations for the control of multi-drug resistant gram-negatives - carbapenem resistant Enterobacteriaceae**	https://www.safetyandquality.gov.au/sites/default/files/migrated/MRGN-Guide-Enterobacteriaceae-PDF-1.89MB.pdf	2013	Infectious diseases	No	No	Not stated
**172. Tumours of the oesophagus and gastro-oesophageal junction structured reporting protocol**	https://www.rcpa.edu.au/getattachment/430d5aea-a8c0-4364-8943-970bb0716c56/Protocol-oesophageal-and-GOJ-cancers.aspx	2013	Pathology	No	No	Not stated
**173. Guidelines for the diagnosis and treatment of malignant pleural mesothelioma**	https://adri.org.au/wp-content/uploads/2018/08/Guidelines-for-the-diagnosis-and-treatment-of-malignant-pleural-mesothelioma.pdf	2013	Public health	Yes	No	Other
**174. Guidelines for the use of support pessaries in the management of pelvic organ prolapse**	https://continence.my.salesforce.com/sfc/p/#A0000000KUc9/a/0f000000Hc5p/odCtTuGR7zC5N0I92XVwNYvJLoaEz1G9DAnzs8puzJM	2012	Urology	No	No	Other
**175. Therapeutic guidelines cardiovascular version 6**	https://www.tg.org.au/the-organisation/expert-groups-2/#Emergency%20Medicine,%20Toxicology%20&%20Wilderness,%20Toxicology%20&%20Toxinology%20expert%20groups	2012	Cardiology	No	No	Not stated
**176. Australian diabetes foot network - management of diabetes-related foot ulceration - a clinical update**	https://www.mja.com.au/system/files/issues/197_04_200812/ber10347_fm.pdf	2012	Endocrinology	No	no	Other

Guideline name	URL	Year	Health topic	NHMRC-approved	GRADE	Funding source
**177. Patient blood management guidelines module 4. Critical care**	https://www.blood.gov.au/system/files/documents/20180424-Module-4.pdf	2012	Haematology	Yes	No	Other
**178. Chronic kidney disease and measurement of albuminuria and proteinuria - a position statement**	https://www.mja.com.au/system/files/issues/197_04_200812/joh11468_web_fm.pdf	2012	Nephrology	No	No	Not stated
**179. Therapeutic guidelines toxicology and wilderness version 2**	https://www.tg.org.au/the-organisation/expert-groups-2/#Emergency%20Medicine,%20Toxicology%20&%20Wilderness,%20Toxicology%20&%20Toxinology%20expert%20groups	2012	Toxicology	No	No	Not stated
**180. Infant feeding guidelines. Information for health workers.**	https://www.nhmrc.gov.au/about-us/publications/infant-feeding-guidelines-information-health-workers#block-views-block-file-attachments-content-block-1	2012	Nutrition	Yes	No	Federal government
**181. Recommendations for the prevention of pregnancy-associated venous thromboembolism**	https://obgyn.onlinelibrary.wiley.com/doi/pdfdirect/10.1111/j.1479-828X.2011.01357.x	2012	Women's health	No	No	Other
**182. Adaptation of the kdigo clinical practice guideline for the care of kidney transplant recipients**	https://onlinelibrary.wiley.com/doi/pdfdirect/10.1111/j.1440-1797.2011.01559.x	2011	Nephrology	No	Yes	Not stated
**183. Aha consensus-based nursing guidelines for the care of patients with hepatitis b, hepatitis c, advanced liver disease and hepatocellular carcinoma**	https://www.aph.gov.au/DocumentStore.ashx?id=af9d0ba5-3f2a-41a6-8ce5-74bdd8fb3168&subId=303639	2012	Infectious diseases	No	No	Other
**184. Clinical practice guidelines antenatal care - module 1**	https://consultations.health.gov.au/phd-tobacco/clinical-practice-guidelines-antenatal-care-module/supporting_documents/ANC_Guidelines_Mod1FINAL%20D13871243.PDF	2012	Women's health	Yes	Yes	Federal government
**185. Guidelines for routine glucose control in hospital**	https://diabetessociety.com.au/documents/ADSGuidelinesforRoutineGlucoseControlinHospitalFinal2012.pdf	2012	Endocrinology	No	No	Not stated
**186. Management of tuberculosis - a handbook for clinicians**	https://researchonline.jcu.edu.au/51444/1/VIDSTBMx_WebVersion%281%29.pdf	2012	Infectious diseases	No	No	Other
**187. Clinical practice guideline for the management of borderline personality disorder**	https://www.nhmrc.gov.au/about-us/publications/clinical-practice-guideline-borderline-personality-disorder#block-views-block-file-attachments-content-block-1	2012	Psychiatry	Yes	No	Federal government
**188. Patient blood management guidelines module 2. Perioperative**	https://www.blood.gov.au/system/files/documents/pbm-module-2.pdf	2012	Haematology	Yes	No	Other
**189. The Australian guideline for prevention, diagnosis and management of acute rheumatic fever and rheumatic heart disease**	http://www.apsu.org.au/assets/past-studies/www.rhdaustralia.org.au-sites-default-files-guideline-0.pdf	2012	Cardiology	No	No	Federal government
**190. Vitamin D and health in adults in Australia and New Zealand - a position statement**	https://www.mja.com.au/system/files/issues/196_11_180612/now10301_web_fm.pdf	2012	Endocrinology	No	No	Other
**191. Patient blood management guidelines module 3. Medical**	https://www.blood.gov.au/system/files/documents/20180424-PBM-Module3.pdf	2012	Haematology	Yes	No	Other

Guideline name	URL	Year	Health topic	NHMRC-approved	GRADE	Funding source
**192. Invasive breast cancer structured reporting protocol**	https://www.rcpa.edu.au/getattachment/9c857cb6-6878-4004-bf8a-37761873cf13/Protocol-invasive-breast-cancer.aspx	2012	Pathology	No	No	Other
**193. National guide to a preventive health assessment for Aboriginal and Torres Strait Islander people**	https://researchonline.jcu.edu.au/40411/12/40411%20Couzos%20et%20al%202012%20short%20version.pdf	2012	Public health	No	No	Federal government
**194. Guidelines for preventive activities in general practice (8th edition)**	https://www.racgp.org.au/download/Documents/Guidelines/Redbook8/redbook8.pdf	2012	General practice	No	No	Not stated
**195. Colorectal cancer structured reporting protocol**	https://confluence.ihtsdotools.org/download/attachments/57808224/2nd-Edition-Colorectal-Protocol-v2-5.pdf?api=v2	2012	Pathology	No	No	Not stated
**196. Clinical practice guidelines for the treatment of lung cancer**	https://wiki.cancer.org.au/australiawiki/index.php?title=Guidelines:Lung_cancer/Guideline_development_process&oldid=47792	2012	Cancer	No	No	Other
**197. Australian and New Zealand Faculty of Radiation Oncology genito-urinary group 2011 consensus guidelines for curative radiotherapy for urothelial carcinoma of the bladder**	https://www.ranzcr.com/documents/4883-australian-new-zealand-faculty-of-radiation-oncology-genito-urinary-group-2011-consensus-guidelines-for-curative-radiotherapy-for-urothelial-carcinoma-of-the-bladder-2012/file	2012	Cancer	No	No	Not stated
**198. Guidelines for the management of absolute cardiovascular disease risk**	https://informme.org.au/Guidelines/Guidelines-for-the-assessment-and-management-of-absolute-CVD-risk	2012	Cardiology	Yes	No	Federal government
**199. Therapeutic guidelines management guidelines developmental disability version 3**	https://www.tg.org.au/the-organisation/expert-groups-2/#Development%20Disability%20expert%20groups	2012	Public health	No	No	Not stated
**200. Therapeutic guidelines oral and dental version 2**	https://www.tg.org.au/the-organisation/expert-groups-2/#Oral%20&%20Dental%20expert%20groups	2012	Dentistry	No	No	Not stated
**201. Practice guidelines for treatment of complex trauma and trauma informed care and service delivery**	https://www.childabuseroyalcommission.gov.au/sites/default/files/IND.0521.001.0001.pdf	2012	Psychiatry	No	No	Federal government
**202. Australian consensus guidelines for the management of neutropenic fever in adult cancer patients**	https://onlinelibrary.wiley.com/doi/epdf/10.1111/j.1445-5994.2010.02338.x	2011	Infectious diseases	No	No	Other
**203. Paediatric empyema thoracis - recommendations for management**	https://www.thoracic.org.au/journal-publishing/command/download_file/id/24/filename/PaediatricEmpyemaThoracisPositionStatementTSANZFINAL.pdf	2011	Respiratory	No	Yes	Not stated
**204. Central nervous system tumour structured reporting protocol (1st edition, 2011)**	https://www.rcpa.edu.au/getattachment/8f65e28f-3377-46de-b27d-b39a44484fb1/Protocol-central-nervous-system-tumours.aspx	2011	Pathology	No	No	Not stated
**205. Recommendations for clinical care guidelines on the management of otitis media in Aboriginal and Torres Strait Islander populations (updated 2010)**	http://www.earandhearinghealth.org.au/file/1562	2011	Public health	No	No	Federal government
**206. Guidelines for the administration of blood products**	https://www.sbp.com.br/fileadmin/user_upload/pdfs/Guidelines_Administration_Blood_Products_2nd.pdf	2011	Haematology	No	No	Not stated
**207. Prevention, identification and management of foot complications in diabetes**	https://baker.edu.au/-/media/documents/impact/diabetes-foot-guidelines/baker-institute-foot-complications-full-guideline.pdf	2011	Endocrinology	Yes	No	Federal government

Guideline name	URL	Year	Health topic	NHMRC-approved	GRADE	Funding source
**208. Australian and New Zealand clinical practice guideline for prevention and management of venous leg ulcers**	https://www.nzwcs.org.nz/images/luag/2011_awma_vlug.pdf	2011	Dermatology	Yes	No	Not stated
**209. Soft tissue tumour resection structured reporting protocol (1st edition, 2011)**	https://www.rcpa.edu.au/getattachment/a3c04ea0-3f34-48a3-86a6-f5bc18345f12/Protocol-Soft-tissue-tumour-resections.aspx	2011	Pathology	No	No	Not stated
**210. Recommendations for staging and managing the axilla in early (operable) breast cancer**	https://www.canceraustralia.gov.au/sites/default/files/publications/maxg-recommendations-for-staging-and-managing-the-axilla-in-early-operable-breast-cancer_504af02f1d112.pdf	2011	Cancer	No	No	Not stated
**211. Evidence-based guidelines to reduce the need for restrictive practices in the disability sector**	https://www.psychology.org.au/getmedia/c986ad95-d312-4b2c-89da-3157b215f118/Restrictive-Practices-Guidelines-for-Psychologists.pdf	2011	Psychiatry	No	No	Not stated
**212. Renal parenchymal malignancy (renal cell carcinoma) structured reporting protocol (1st edition 2011)**	http://docplayer.net/18320658-Renal-parenchymal-malignancy-renal-cell-carcinoma-structured-reporting-protocol-1st-edition-2011.html	2011	Pathology	No	No	Not stated
**213. Clinical practice guidelines for the treatment and management of endometrial cancer**	https://wiki.cancer.org.au/australia/Guidelines:Endometrial_cancer/Treatment/Early_stage	2011	Cancer	No	No	Not stated
**214. Evidence based practice guidelines for the dietetic management of adults with pressure injuries**	https://www.aci.health.nsw.gov.au/__data/assets/pdf_file/0004/388237/13.-Trans-Tasman-Dietetic-Wound-Care-Group-Pressure-Injury-Guidelines-2011.pdf	2011	Nutrition	No	No	Not stated
**215. Psychosocial management of AYAs diagnosed with cancer - guidance for health professionals**	https://wiki.cancer.org.au/australia/COSA:Psychosocial_management_of_AYA_cancer_patients	2011	Cancer	No	No	Federal government
**216. Patient blood management guidelines module 1. Critical bleeding massive transfusion**	https://www.blood.gov.au/system/files/documents/Module%201.pdf	2011	Haematology	No	No	Other
**217. Therapeutic guidelines ulcer and wound management**	https://www.tg.org.au/the-organisation/expert-groups-2/#Gastrointestinal%20expert%20groups	2012	Dermatology	No	No	Not stated
**218. Fertility preservation for AYAs diagnosed with cancer - guidance for health professionals**	https://wiki.cancer.org.au/australiawiki/index.php?title=COSA:AYA_cancer_fertility_preservation&oldid=12607	2011	Cancer	No	No	Federal government
**219. Therapeutic guidelines gastrointestinal version 5**	https://www.tg.org.au/the-organisation/expert-groups-2/#Gastrointestinal%20expert%20groups	2011	Gastroenterology	No	No	Not stated
**220. A consensus statement for safety monitoring guidelines of treatments for major depressive disorder**	https://journals.sagepub.com/doi/pdf/10.3109/00048674.2011.595686	2011	Psychiatry	No	No	Other
**221. National evidence based clinical care guidelines for type 1 diabetes in children, adolescents and adults**	https://diabetessociety.com.au/documents/Type1guidelines14Nov2011.pdf	2011	Endocrinology	Yes	No	Federal government

Guideline name	URL	Year	Health topic	NHMRC-approved	GRADE	Funding source
**222. Evidence based practice guidelines for the nutritional management of adult patients with head and neck cancer**	https://wiki.cancer.org.au/australia/COSA:Head_and_neck_cancer_nutrition_guidelines/Introduction	2011	Cancer	No	No	Other
**223. Evidence-based guideline for the assessment and management of polycystic ovary syndrome**	https://www.mja.com.au/system/files/issues/195_06_190911/tee10915_fm.pdf	2011	Women's Health	Yes	No	Federal government
**224. Clinical practice guidelines. Depression in adolescents and young adults (February 2011)**	http://www.24hmb.com/voimages/web_image/upload/file/20140614/20851402752241427.pdf	2011	Psychiatry	No	No	Other
**225. Testicular tumours structured reporting protocol (1st edition 2011)**	https://studylib.net/doc/12265219/testicular-tumours-structured-reporting-protocol--1st-edi...	2011	Pathology	No	No	Not stated
**226. Consensus-based clinical practice guidelines for the management of volatile substance use in Australia**	https://vsu.mhc.wa.gov.au/media/1189/nhmrc-1.pdf	2011	Public health	Yes	No	Federal government
**227. Australasian contact tracing manual 4th edition 2010**	https://ashm.blob.core.windows.net/ashmpublic/CTM_2010.pdf	2010	Infectious diseases	No	No	Federal government
**228. Australian and New Zealand Faculty of Radiation Oncology genito-urinary group: 2010 consensus guidelines for definitive external beam radiotherapy for prostate carcinoma**	https://www.ranzcr.com/documents-download/professional-documents/guidelines/4882-australian-new-zealand-faculty-of-radiation-oncology-genito-urinary-group-2010-consensus-guidelines-for-definitive-external-beam-radiotherapy-for-prostate-carcinoma/file	2010	Cancer	No	No	Cancer
**229. Therapeutic guidelines: palliative care, version 3**	https://www.tg.org.au/the-organisation/expert-groups-2/#Palliative%20Care%20expert%20groups	2010	Palliative care	No	No	Not stated
**230. Palliative care in the neonatal nursery. Guidelines for neonatal nurses in Australia**	https://www.acnn.org.au/resources/clinical-guidelines/G3-Palliative-care-in-the-neonatal-nursery.pdf	2010	Paediatrics	No	No	Not stated
**231. Clinical guideline for the prevention and treatment of osteoporosis in postmenopausal women and older men**	https://www.racgp.org.au/download/documents/Guidelines/Musculoskeletal/racgp_osteo_guideline.pdf	2010	General practice	Yes	No	NHMRC
**232. Type 2 diabetes - kidney disease**	https://onlinelibrary.wiley.com/toc/14401797/15/s1	2010	Nephrology	No	No	Federal government
**233. Guidelines for patient selection and performance of carotid artery stenting**	https://onlinelibrary.wiley.com/doi/pdfdirect/10.1111/j.1445-2197.2010.05330.x	2010	Cardiology	No	No	Not stated
**234. Cicada - cough in children and adults - diagnosis and assessment. Australian cough guidelines summary statement**	https://www.mja.com.au/system/files/issues/192_05_010310/gib10754_fm.pdf	2010	Respiratory	No	Yes	Not stated
**235. Clinical practice guidelines for the psychosocial and bereavement support of family caregivers and palliative care patients**	https://engonetcpc.blob.core.windows.net/assets/uploads/files/Assets/CPG%20Psychosocial%20bereavement%20suport(1).pdf	2010	Palliative care	No	No	Other
**236. Recommendations for bariatric surgery in adolescents in Australia and New Zealand**	https://www.racp.edu.au/docs/default-source/advocacy-library/recommendations-for-bariatric-surgery-in-adolescents.pdf	2010	Paediatrics	No	No	Not stated

Guideline name	URL	Year	Health topic	NHMRC-approved	GRADE	Funding source
**237. Antenatal magnesium sulphate prior to preterm birth for neuroprotection of the fetus, infant and child 2010**	https://www.sahmri.org/m/downloads/cp128_mag_sulphate_child.pdf	2010	Women's health	Yes	No	Not stated
**238. Therapeutic guidelines rheumatology version 2**	https://www.tg.org.au/the-organisation/expert-groups-2/	2010	Rheumatology	No	No	Not stated
**239. NHMRC guidelines for the screening, prognosis, diagnosis, management and prevention of glaucoma**	https://www.nhmrc.gov.au/about-us/publications/guidelines-screening-prognosis-diagnosis-management-and-prevention-glaucoma#block-views-block-file-attachments-content-block-1	2010	Ophthalmolog y	No	No	Federal government
**240. Australian guidelines for the prevention and control of infection in healthcare**	https://www.nhmrc.gov.au/about-us/publications/Australian-guidelines-prevention-and-control-infection-healthcare-2010	2010	Public health	No	No	Federal government
**241. Acute pain management: scientific evidence (third edition 2010)**	https://airr.anzca.edu.au/anzcajspui/bitstream/11055/928/1/Acute%20pain%20management%20-%20scientific%20evidence%20-%20third%20edition.pdf	2010	Anaesthetics	Yes	No	Not stated
**242. Clinical practice guideline for the management of locally advanced and metastatic prostate cancer**	https://wiki.cancer.org.au/Australia/Guidelines:Prostate_cancer/Management/Locally_advanced_and_metastatic/Working_party_members_and_contributors	2010	Cancer	No	No	Not stated
**243. Tumours of hematopoietic and lymphoid tissue structured reporting protocol (1st edition)**	https://www.rcpa.edu.au/getattachment/f32439ee-36ed-4f19-a9cf-a7759e2a60a6/Protocol-haematopoietic-and-lymphoid-tissue.aspx	2010	Pathology	No	No	Not stated
**244. Recommendations for use of chemotherapy for the treatment of advanced breast cancer**	https://www.cancerAustralia.gov.au/sites/default/files/publications/cmag-chemotherapy-guidelines-for-advanced-breast-cancer_504af030defd3.pdf	2010	Cancer	No	No	Other
**245. Clinical guidelines for stroke management 2010**	https://www.pedro.org.au/wp-content/uploads/CPG_stroke.pdf	2010	Neurology	Yes	No	Federal governemen t
**246. Treatment for osteoporosis in Australian residential aged care facilities: consensus recommendations for fracture prevention**	https://www.mja.com.au/journal/2010/193/3/treatment-osteoporosis-Australian-residential-aged-care-facilities-consensus	2010	Endocrinology	No	No	Other
**Fewer than ten guideline panel members**						
**247. Evidenced-based clinical practice guideline for management of newborn pain**	https://painsa.org.za/wp-content/uploads/2015/04/journal-guideline-2010.pdf	2010	Paediatrics	No	No	Federal government
**248. Management of pancreatic exocrine insufficiency: Australasian pancreatic club recommendations**	http://mja.com.au/journal/2010/193/8/management-pancreatic-exocrine-insufficiency-australasian-pancreatic-club#:~:text=Pancreatic%20enzyme%20replacement%20therapy%20is,units%20of%20lipase%20per%20meal.	2010	Endocrinology	No	No	Not stated
**249. Nutrition in kidney transplant recipients**	https://onlinelibrary.wiley.com/toc/14401797/2010/15/s1	2010	Nephrology	No	No	Other
**250. Clinical practice guidelines for the management of acute limb compartment syndrome following trauma**	https://onlinelibrary.wiley.com/doi/pdfdirect/10.1111/j.1445-2197.2010.05213.x	2010	Surgery	No	No	None
**251. Renovascular disease**	https://onlinelibrary.wiley.com/toc/14401797/2010/15/s1	2010	Nephrology	No	No	Not stated

Guideline name	URL	Year	Health topic	NHMRC-approved	GRADE	Funding source
**252. Problematic alcohol and other drug use in the Australian aviation sector - comprehensive assessment guidelines**	https://www.casa.gov.au/file/169836/download?token=uSHjvPIZ	2010	Aviation medicine	No	No	Not stated
**253. Nursing clinical practice guidelines to improve care for people undergoing percutaneous coronary interventions**	https://www.academia.edu/18894560/Nursing_clinical_practice_guidelines_to_improve_care_for_people_undergoing_percutaneous_coronary_interventions	2011	Cardiology	No	No	Other
**254. Australasian society for infectious diseases guidelines for the diagnosis and treatment of clostridium difficile infection**	https://www.mja.com.au/system/files/issues/194_07_040411/che10934_fm.pdf	2011	Infectious diseases	No	No	Not stated
**255. Diagnostic approach to fatigue in primary care**	https://www.tg.org.au/the-organisation/expert-groups-2/#Gastrointestinal%20expert%20groups	2014	General practice	No	No	Not stated
**256. Bone and metabolic health in patients with non-metastatic prostate cancer who are receiving androgen deprivation therapy**	https://www.mja.com.au/system/files/issues/194_06_210311/gro10289_fm.pdf	2011	Endocrinology	Yes	No	Federal government
**257. Guidelines for screening, assessment and treatment in problem gambling**	https://www.mja.com.au/journal/2011/195/11/Australian-guideline-treatment-problem-gambling-abridged-outline	2011	Gambling and addiction	Yes	No	Not stated
**258. Guidelines for the prevention, detection and management of chronic heart failure (updated October 2011)**	https://www.csanz.edu.au/documents/guidelines/clinical_practice/2011_HF_CSANZ_Chronic_Heart_Failure.pdf	2011	Cardiology	No	No	Not stated
**259. ASID (HICSIG)/AICA position statement - preventing catheter-associated urinary tract infections in patients**	https://www.publish.csiro.au/HI/pdf/HI11007	2011	Infectious disease	No	No	Not stated
**260. Guidelines for the diagnosis and management of catecholaminergic polymorphic ventricular tachycardia**	https://www.csanz.edu.au/wp-content/uploads/2013/11/Diagnosis-and-Management-of-CPVT.pdf	2011	Cardiology	No	No	Not stated
**261. Vascular access**	https://www.cariguidelines.org/guidelines/dialysis/vascular-access/	2012	Nephrology	No	No	Not stated
**262. How we use recombinant activated factor VII in patients with haemophilia a or b complicated by inhibitors**	https://onlinelibrary.wiley.com/doi/pdfdirect/10.1111/j.1445-5994.2012.02942.x	2012	Haematology	No	No	Other
**263. Asthma and allergy. An information paper for health professionals**	https://d8z57tiamduo7.cloudfront.net/resources/asthma-allergy-hp.pdf	2012	Respiratory	No	No	Federal government
**264. Reducing risk in heart disease - an expert guide to clinical practice for secondary prevention of coronary heart disease (updated 2012)**	https://www.csanz.edu.au/wp-content/uploads/2014/12/2012_HF_CSANZ_Reducing_Risk_in_Heart_Disease.pdf	2012	Cardiology	No	No	Not stated
**265. The role of HBA1C in the diagnosis of diabetes mellitus in Australia**	https://www.mja.com.au/system/files/issues/197_04_200812/dem10988_web_fm.pdf	2012	Endocrinology	No	Yes	Not stated
**266. Vitamin D and health in adults in Australia and New Zealand - a position statement**	https://www.mja.com.au/system/files/issues/196_11_180612/now10301_web_fm.pdf	2012	Endocrinology	No	No	Not stated
**267. Peri-operative diabetes management guidelines**	https://diabetessociety.com.au/documents/PerioperativeDiabetesManagementGuidelinesFINALCleanJuly2012.pdf	2012	Endocrinology	No	No	Not stated

Guideline name	URL	Year	Health topic	NHMRC-approved	GRADE	Funding source
**268. Evidence-based approaches to the management of cognitive and behavioral impairments following pediatric brain injury**	https://www-proquest-com.ap1.proxy.openathens.net/docview/1141684293?accountid=12528	2012	Paediatrics	No	No	Not stated
**269. Recipient assessment for transplantation**	https://onlinelibrary.wiley.com/doi/pdfdirect/10.1111/nep.12068	2013	Nephrology	No	Yes	Not stated
**270. ANZICS statement on death and organ donation (edition 3.2)**	https://csds.qld.edu.au/sdc/Provectus/ELI/Module%202%20-%20Organ%20donation%20after%20brain%20death/files/ANZICS%20Statement%20on%20%20Death%20and%20Organ%20Donation%20Edition%203.2%20(3).pdf	2013	Intensive care	No	No	Federal government
**271. Guidelines for the diagnosis and management of familial hypercholesterolemia**	https://www.csanz.edu.au/wp-content/uploads/2013/12/Familial_Hypercholesterolemia_2013.pdf	2013	Cardiology	No	No	Not stated
**272. National Heart Foundation of Australia consensus statement on catheter ablation as a therapy for atrial fibrillation**	https://www.mja.com.au/system/files/issues/198_01_210113/kal10929_web%20version_fm.pdf	2013	Cardiology	No	No	Other
**273. Australian and New Zealand national evidence-based recommendations for the investigation and follow-up of undifferentiated peripheral inflammatory arthritis. An integration of systematic literature research and rheumatological expert opinion**	https://onlinelibrary.wiley.com/doi/pdfdirect/10.1111/1756-185X.12189	2013	Rheumatology	No	No	Other
**274. Malignant salivary gland neoplasms structured reporting protocol (1st edition 2013)**	https://studylib.net/doc/12256181/malignant-salivary-gland-neoplasms-structured-reporting-p...	2013	Pathology	No	No	Not stated
**275. Skin prick testing for the diagnosis of allergic disease**	https://www.allergy.org.au/images/stories/pospapers/ASCIA_HP_SPT_Manual_May2013.pdf	2013	Immunology	No	No	Not stated
**276. Guidelines for the diagnosis and management of hypertrophic cardiomyopathy**	https://www.csanz.edu.au/wp-content/uploads/2014/01/Hyertrophic-Cardiomyopathy-2013.pdf	2013	Cardiology	No	No	Not stated
**277. Dialysis adequacy - haemodialysis**	https://onlinelibrary.wiley.com/doi/pdfdirect/10.1111/nep.12096	2013	Nephrology	No	Yes	Not stated
**278. Guideline for the management of major regional anaesthesia**	https://www.anzca.edu.au/getattachment/159a8905-b558-480b-82d7-79a653ff83a0/PS03-Guideline-for-the-management-of-major-regional-analgesia	2014	Intensive care	No	No	Not stated
**279. Clinical guiding principles for sick day management of adults with type 1 and type 2 diabetes**	http://www.klmc.net.au/pdf/adea-sick-day-management-guidelines.pdf	2014	Endocrinology	O	No	None
**280. Diagnosis and treatment of urinary tract infection in children**	https://onlinelibrary.wiley.com/doi/pdfdirect/10.1111/nep.12349	2014	Nephrology	No	Yes	Not stated
**281. New oral anticoagulants - a practical guide on prescription, laboratory testing and peri-procedural bleeding management**	https://onlinelibrary.wiley.com/doi/pdfdirect/10.1111/imj.12448	2014	Haematology	No	No	None
**282. Clinical guiding principles for sick day management of adults with type 1 and type 2 diabetes**	http://www.klmc.net.au/pdf/adea-sick-day-management-guidelines.pdf	2014	Endocrinology	No	Yes	Other

Guideline name	URL	Year	Health topic	NHMRC-approved	GRADE	Funding source
**283. The conservative management of renal trauma - a literature review and practical clinical guideline from Australia and New Zealand**	https://bjui-journals.onlinelibrary.wiley.com/doi/epdf/10.1111/bju.12902	2014	Urology	No	No	Not stated
**284. Aphasia rehabilitation best practice statements 2014. Comprehensive supplement to the Australian aphasia rehabilitation pathway**	http://www.aphasiapathway.com.au/flux-content/aarp/pdf/2014-COMPREHENSIVE-FINAL-01-10-2014-1.pdf	2014	Neurology	No	No	NHMRC
**285. 2011 update to National Heart Foundation of Australia and Cardiac Society of Australia and New Zealand guidelines for the prevention, detection and management of chronic heart failure in Australia, 2006**	https://onlinelibrary.wiley.com/doi/abs/10.5694/j.1326-5377.2011.tb03031.x	2011	Cardiology	No	No	Not stated
**286. Consensus standards for the care of children and adolescents in Australian health services**	https://onlinelibrary.wiley.com/doi/abs/10.5694/j.1326-5377.2011.tb04172.x	2011	Paediatrics	No	No	Not stated
**287. Australian guideline for treatment of problem gambling: an abridged outline**	https://onlinelibrary.wiley.com/doi/abs/10.5694/mja11.11088	2011	Gambling and addiction	Yes	no	Not stated
**288. The consensus statement on hip surveillance for children with cerebral palsy: Australian standards of care**	https://content.iospress.com/articles/journal-of-pediatric-rehabilitation-medicine/prm00174	2011	Paediatrics	No	No	Not stated
**289. Alcohol and cancer: a position statement from Cancer Council Australia**	https://onlinelibrary.wiley.com/doi/abs/10.5694/j.1326-5377.2011.tb03067.x	2011	Cancer	No	No	Not stated
**290. Evidence-based asthma management in children--what's new?**	https://onlinelibrary.wiley.com/doi/abs/10.5694/j.1326-5377.2011.tb03025.x	2011	Cardiology	No	No	Not stated
**291. Reducing risk in heart disease - an expert guide to clinical practice for secondary prevention of coronary heart disease**	https://regroup-production.s3.amazonaws.com/documents/ReviewReference/350659677/2012-Reducing%20risk%20in%20heart%20disease%20-%20an%20exper.pdf?response-content-type=application%2Fpdf&X-Amz-Algorithm=AWS4-HMAC-SHA256&X-Amz-Credential=AKIAYSFKCAWY23RWESRS%2F20220911%2Fus-east-1%2Fs3%2Faws4_request&X-Amz-Date=20220911T140317Z&X-Amz-Expires=604800&X-Amz-SignedHeaders=host&X-Amz-Signature=ea3d928865758ff84e684f3238267c13cdfbdabfa8687846adc2c18f08a50e34	2012	Cardiology	No	No	Not stated
**292. Vitamin D and health in adults in Australia and New Zealand: a position statement**	https://onlinelibrary.wiley.com/doi/10.5694/mja11.10301	2012	Osteoporosis	No	No	Not stated
**293. Vitamin D and health in pregnancy, infants, children and adolescents in Australia and New Zealand. A position statement**	https://onlinelibrary.wiley.com/doi/pdfdirect/10.5694/mja11.11592	2013	Osteoporosis	No	No	None
**294. Early chronic kidney disease**	https://onlinelibrary.wiley.com/doi/pdfdirect/10.1111/nep.12052	2013	Nephrology	No	No	Not stated

Guideline name	URL	Year	Health topic	NHMRC-approved	GRADE	Funding source
**295. The ANZICS statement on death and organ donation (edition 3.2)**	https://regroup-production.s3.amazonaws.com/documents/ReviewReference/350659419/2013-The%20ANZICS%20statement%20on%20death%20and%20organ%20d.pdf?response-content-type=application%2Fpdf&X-Amz-Algorithm=AWS4-HMAC-SHA256&X-Amz-Credential=AKIAYSFKCAWY23RWESRS%2F20220911%2Fus-east-1%2Fs3%2Faws4_request&X-Amz-Date=20220911T135928Z&X-Amz-Expires=604800&X-Amz-SignedHeaders=host&X-Amz-Signature=fcd45323ee6064c2bbd13464ea4792699b5d961713c0f2675b45807a348e2b28	2013	Intensive care	No	No	Federal government
**296. An update of consensus guidelines for warfarin reversal**	https://www.mja.com.au/system/files/issues/tra10614_web_fm_0.pdf	2013	Haematology	No	Yes	Other
**297. The development of a clinical practice guideline to improve sleep in intensive care patients: a solution focused approach**	https://www.sciencedirect.com/science/article/abs/pii/S0964339714000378?via%3Dihub	2014	Intensive care	Nio	No	Other
**298. Australasian Society for parenteral and Enteral Nutrition guidelines for supplementation of trace elements during parenteral nutrition**	https://www.airitilibrary.com/Publication/alDetailedMesh?DocID=09647058-201303-PP201303130005-PP201303130005-1-20-0075	2014	Nutrition	No	No	Other
**299. Clinical Oncology Society of Australia position statement on the use of complementary and alternative medicine by cancer patients**	https://onlinelibrary.wiley.com/doi/10.1111/ajco.12227	2014	Cancer	No	No	Not stated
**300. Developing the Australasian Hepatology Association's consensus-based guidelines for the nursing care of patients with liver disease**	https://regroup-production.s3.amazonaws.com/documents/ReviewReference/350657737/Richmond-2014-Developing%20the%20Australasian%20Hepa.pdf?response-content-type=application%2Fpdf&X-Amz-Algorithm=AWS4-HMAC-SHA256&X-Amz-Credential=AKIAYSFKCAWY23RWESRS%2F20220911%2Fus-east-1%2Fs3%2Faws4_request&X-Amz-Date=20220911T135606Z&X-Amz-Expires=604800&X-Amz-SignedHeaders=host&X-Amz-Signature=bfc26b61cff67e634be7c0939ff92c6d16bccd040a51cbc1980b5bbffc997220	2014	Gastroenterology	No	No	Other
**301. Guidance on the clinical treatment of people with anorexia nervosa, bulimia nervosa, binge eating disorder and ARFID**	https://regroup-production.s3.amazonaws.com/documents/ReviewReference/350659639/2014-Guidance%20on%20the%20clinical%20treatment%20of%20peo.pdf?response-content-type=application%2Fpdf&X-Amz-Algorithm=AWS4-HMAC-SHA256&X-Amz-Credential=AKIAYSFKCAWY23RWESRS%2F20220911%2Fus-east-1%2Fs3%2Faws4_request&X-Amz-Date=20220911T134225Z&X-Amz-Expires=604800&X-Amz-SignedHeaders=host&X-Amz-Signature=4f7379921d53638efe9d677a63625315e7591d9dca6922a067ea6586b773147e	2014	Psychiatry	No	No	Other

Guideline name	URL	Year	Health topic	NHMRC-approved	GRADE	Funding source
**302. A new blood glucose management algorithm for type 2 diabetes: a position statement of the Australian Diabetes Society**	https://onlinelibrary.wiley.com/doi/abs/10.5694/mja14.01187	2014	Endocrinology	No	No	Not stated
**303. Cardiovascular disease in patients with chronic kidney disease**	https://regroup-production.s3.amazonaws.com/documents/ReviewReference/350659974/2014-Cardiovascular%20disease%20in%20patients%20with%20c.pdf?response-content-type=application%2Fpdf&X-Amz-Algorithm=AWS4-HMAC-SHA256&X-Amz-Credential=AKIAYSFKCAWY23RWESRS%2F20220911%2Fus-east-1%2Fs3%2Faws4_request&X-Amz-Date=20220911T134316Z&X-Amz-Expires=604800&X-Amz-SignedHeaders=host&X-Amz-Signature=4695b774abd346898967bb8a099871c58bbfa19d93a17e9c92f242ba5352da21	2013	Nephrology	No	No	Not stated
**304. Diagnosis & treatment of urinary tract infection in children**	https://regroup-production.s3.amazonaws.com/documents/ReviewReference/350659975/2014-Diagnosis%20%26%20Treatment%20of%20Urinary%20Tract%20In.pdf?response-content-type=application%2Fpdf&X-Amz-Algorithm=AWS4-HMAC-SHA256&X-Amz-Credential=AKIAYSFKCAWY23RWESRS%2F20220911%2Fus-east-1%2Fs3%2Faws4_request&X-Amz-Date=20220911T134408Z&X-Amz-Expires=604800&X-Amz-SignedHeaders=host&X-Amz-Signature=c97ef06e4e887e5dfa714a3dca58e28494cf13e5ac685b1e88738cefd133489d	2014	Nephrology	No	No	Not stated
**305. KHA-CARI guideline: peritonitis treatment and prophylaxis**	https://regroup-production.s3.amazonaws.com/documents/ReviewReference/350659977/2014-Dialysis%20-%20Peritonitis%20Treatment%20and%20Prop.pdf?response-content-type=application%2Fpdf&X-Amz-Algorithm=AWS4-HMAC-SHA256&X-Amz-Credential=AKIAYSFKCAWY23RWESRS%2F20220911%2Fus-east-1%2Fs3%2Faws4_request&X-Amz-Date=20220911T134558Z&X-Amz-Expires=604800&X-Amz-SignedHeaders=host&X-Amz-Signature=410bcec31fd99e4619ce0d88880116c1dbdd959e1dd8c2d3c2efb97b13059e14	2013	Nephrology	No	No	Not stated
**306. Sedation for cardiological procedures**	https://regroup-production.s3.amazonaws.com/documents/ReviewReference/350659583/2014-Sedation%20for%20Cardiological%20Procedures.pdf?response-content-type=application%2Fpdf&X-Amz-Algorithm=AWS4-HMAC-SHA256&X-Amz-Credential=AKIAYSFKCAWY23RWESRS%2F20220911%2Fus-east-1%2Fs3%2Faws4_request&X-Amz-Date=20220911T134623Z&X-Amz-Expires=604800&X-Amz-SignedHeaders=host&X-Amz-Signature=f04140917cfab3290ef412adc341bffaf98fae08abc1859170bc2cf0b320a0f8	2014	Cardiology	No	No	Not stated

Guideline name	URL	Year	Health topic	NHMRC-approved	GRADE	Funding source
**307. Section 2. Treatment of acute kidney injury**	https://regroup-production.s3.amazonaws.com/documents/ReviewReference/350659979/2014-Section%202.%20Treatment%20of%20acute%20kidney%20inju.pdf?response-content-type=application%2Fpdf&X-Amz-Algorithm=AWS4-HMAC-SHA256&X-Amz-Credential=AKIAYSFKCAWY23RWESRS%2F20220911%2Fus-east-1%2Fs3%2Faws4_request&X-Amz-Date=20220911T134645Z&X-Amz-Expires=604800&X-Amz-SignedHeaders=host&X-Amz-Signature=6c6b7a99eb475308f65738b3c70bfa8848bf32f1cd24dbb6bf280b250ac410f1	2014	nephrology	No	No	Not stated
**308. Section 3. Prevention of acute kidney injury**	https://regroup-production.s3.amazonaws.com/documents/ReviewReference/350659980/2014-Section%203.%20Prevention%20of%20acute%20kidney%20inj.pdf?response-content-type=application%2Fpdf&X-Amz-Algorithm=AWS4-HMAC-SHA256&X-Amz-Credential=AKIAYSFKCAWY23RWESRS%2F20220911%2Fus-east-1%2Fs3%2Faws4_request&X-Amz-Date=20220911T134717Z&X-Amz-Expires=604800&X-Amz-SignedHeaders=host&X-Amz-Signature=e9239925fa3401c9807c1b35cb7a7962dbaf1eed22881178f07a1e8efe52a822	2014	nephrology	No	No	Not stated
**309. Section 4. Contrast induced acute kidney injury**	https://regroup-production.s3.amazonaws.com/documents/ReviewReference/350659981/2014-Section%204.%20Contrast%20induced%20acute%20kidney.pdf?response-content-type=application%2Fpdf&X-Amz-Algorithm=AWS4-HMAC-SHA256&X-Amz-Credential=AKIAYSFKCAWY23RWESRS%2F20220911%2Fus-east-1%2Fs3%2Faws4_request&X-Amz-Date=20220911T134826Z&X-Amz-Expires=604800&X-Amz-SignedHeaders=host&X-Amz-Signature=932875c61199e6e5919f76f4104427016b8e27bd6271135e3d52b01b748b0a23	2014	nephrology	No	No	Not stated
**310. Section 5.1 dialysis interventions for the treatment of acute kidney injury: anticoagulation**	https://regroup-production.s3.amazonaws.com/documents/ReviewReference/350659983/2014-Section%205.1%20Dialysis%20interventions%20for%20th.pdf?response-content-type=application%2Fpdf&X-Amz-Algorithm=AWS4-HMAC-SHA256&X-Amz-Credential=AKIAYSFKCAWY23RWESRS%2F20220911%2Fus-east-1%2Fs3%2Faws4_request&X-Amz-Date=20220911T134948Z&X-Amz-Expires=604800&X-Amz-SignedHeaders=host&X-Amz-Signature=d0c83dfc138c1ab46074ca7ac146825f6ffb10f2dfc892dd4842b0eeaa5d824c	2014	nephrology	No	No	Not stated

Guideline name	URL	Year	Health topic	NHMRC-approved	GRADE	Funding source
**311. Section 1. Definition and classification of acute kidney injury**	https://regroup-production.s3.amazonaws.com/documents/ReviewReference/350659978/2014-Section%201.%20Definition%20and%20classification.pdf?response-content-type=application%2Fpdf&X-Amz-Algorithm=AWS4-HMAC-SHA256&X-Amz-Credential=AKIAYSFKCAWY23RWESRS%2F20220911%2Fus-east-1%2Fs3%2Faws4_request&X-Amz-Date=20220911T135219Z&X-Amz-Expires=604800&X-Amz-SignedHeaders=host&X-Amz-Signature=aaec6f54d16253d4a6d64ae00828b38321981565d7e1ddbd8c573e88c02ef563	2014	nephrology	No	No	Not stated
**312. Section 5.2. Dialysis interventions for the treatment of acute kidney injury**	https://regroup-production.s3.amazonaws.com/documents/ReviewReference/350659984/2014-Section%205.2.%20Dialysis%20interventions%20for%20t.pdf?response-content-type=application%2Fpdf&X-Amz-Algorithm=AWS4-HMAC-SHA256&X-Amz-Credential=AKIAYSFKCAWY23RWESRS%2F20220911%2Fus-east-1%2Fs3%2Faws4_request&X-Amz-Date=20220911T135247Z&X-Amz-Expires=604800&X-Amz-SignedHeaders=host&X-Amz-Signature=3efb7a30af791423c7a9042179d150001225958ea94f574f517be8bace36a8ee	2014	nephrology	No	No	Not stated
**313. CSANZ position statement on sedation for cardiovascular procedures (2014)**	https://linkinghub.elsevier.com/retrieve/pii/S1443950615004217	2014	Cardiology	No	No	Not stated
**314. Consensus recommendations on the use of daylight photodynamic therapy with methyl aminolevulinate cream for actinic keratoses in Australia**	https://onlinelibrary.wiley.com/doi/10.1111/ajd.12354	2015	Dermatology	No	No	Other
**315. Refining the care of patients with pancreatic cancer: the AGITG pancreatic cancer workshop consensus**	https://onlinelibrary.wiley.com/doi/abs/10.5694/mja16.00061	2016	Caner	No	No	Other
**316. Update on the diagnosis and management of inherited aortopathies, including Marfan syndrome**	https://regroup-production.s3.amazonaws.com/documents/ReviewReference/350660230/2016-Inherited%20aortopathies%20including%20Marfan%20S.pdf?response-content-type=application%2Fpdf&X-Amz-Algorithm=AWS4-HMAC-SHA256&X-Amz-Credential=AKIAYSFKCAWY23RWESRS%2F20220911%2Fus-east-1%2Fs3%2Faws4_request&X-Amz-Date=20220911T133110Z&X-Amz-Expires=604800&X-Amz-SignedHeaders=host&X-Amz-Signature=70918d64ee0ca822412458d05b22a55bc3c2d6317464f20693ff106ffd97ad61	2016	Cardiology	No	No	Not stated

Guideline name	URL	Year	Health topic	NHMRC-approved	GRADE	Funding source
**317. Diagnosis and management of familial dilated cardiomyopathy - position statement**	https://regroup-production.s3.amazonaws.com/documents/ReviewReference/350660224/2016-Familial%20Dilated%20Cardiomyopathy.pdf?response-content-type=application%2Fpdf&X-Amz-Algorithm=AWS4-HMAC-SHA256&X-Amz-Credential=AKIAYSFKCAWY23RWESRS%2F20220911%2Fus-east-1%2Fs3%2Faws4_request&X-Amz-Date=20220911T132539Z&X-Amz-Expires=604800&X-Amz-SignedHeaders=host&X-Amz-Signature=a685939b0644ab18de905ae24535e9b012c347c350d1a402d66988a54160b472	2016	Cardiology	No	No	Not stated
**318. Australian and New Zealand Anaesthetic Allergy Group/Australian and New Zealand College of Anaesthetists perioperative anaphylaxis management guidelines**	https://regroup-production.s3.amazonaws.com/documents/ReviewReference/351122822/Australia-2012-Australian%20guideline%20for%20preven.pdf?response-content-type=application%2Fpdf&X-Amz-Algorithm=AWS4-HMAC-SHA256&X-Amz-Credential=AKIAYSFKCAWY23RWESRS%2F20220911%2Fus-east-1%2Fs3%2Faws4_request&X-Amz-Date=20220911T132342Z&X-Amz-Expires=604800&X-Amz-SignedHeaders=host&X-Amz-Signature=582cfeeb330d8628d4d2e65d6706c6f26f4398fb3acf35ff6c95cb7e356ef506	2017	Immunology	No	No	Not stated
**319. Spleen Australia guidelines for the prevention of sepsis in patients with asplenia and hyposplenism in Australia and New Zealand**	https://onlinelibrary.wiley.com/doi/10.1111/imj.13348	2017	Infectious diseases	No	No	None
**320. Australian Institute of Sport and Australian Medical Association position statement on concussion in sport**	https://onlinelibrary.wiley.com/doi/abs/10.5694/mja16.00741	2017	Exercise medicine	No	No	Federal government
**321. Clinical practice guidelines for the diagnosis and management of melanoma: melanomas that lack classical clinical features**	https://onlinelibrary.wiley.com/doi/abs/10.5694/mja17.00123	2017	Cancer	No	No	Other
**322. Cardiac Society of Australia and New Zealand position statement executive summary: coronary artery calcium scoring**	https://onlinelibrary.wiley.com/doi/abs/10.5694/mja16.01134	2017	Cardiology	No	No	Not stated
**323. Updated evidence-based clinical practice guidelines for the diagnosis and management of melanoma: definitive excision margins for primary cutaneous melanoma**	https://onlinelibrary.wiley.com/doi/abs/10.5694/mja17.00278	2018	Cancer	No	No	Other
**324. Australian standards of care and treatment guidelines for transgender and gender diverse children and adolescents**	https://onlinelibrary.wiley.com/doi/abs/10.5694/mja18.00286	2018	Paediatrics	No	No	Not stated
**325. Position statement: a clinical approach to the management of adult non-neurogenic overactive bladder**	https://onlinelibrary.wiley.com/doi/abs/10.5694/mja16.01097	2018	Urology	No	No	Not stated

Guideline name	URL	Year	Health topic	NHMRC-approved	GRADE	Funding source
**326. ASCO/CAP 2018 breast cancer her2 testing guidelines: summary of pertinent recommendations for practice in Australia**	https://www.pathologyjournal.rcpa.edu.au/article/S0031-3025(18)30615-9/fulltext	2019	Pathology	No	No	Not stated
**327. Australian infection control in endoscopy consensus statements on carbapenemase-producing Enterobacteriaceae**	https://onlinelibrary.wiley.com/doi/10.1111/jgh.14511	2019	Gastroenterology	No	No	Not stated
**328. The Australasian Society of Clinical Immunology and allergy infant feeding for allergy prevention guidelines**	https://onlinelibrary.wiley.com/doi/abs/10.5694/mja2.12102	2019	Immunology	No	Yes	Not stated
**329. Updated Australian consensus statement on management of inherited bleeding disorders in pregnancy**	https://onlinelibrary.wiley.com/doi/full/10.5694/mja2.50123	2019	Haematology	No	No	Not stated
**330. Common clinical conditions in aged care (silver book)**	https://regroup-production.s3.amazonaws.com/documents/ReviewReference/350660124/2019-Common%20clinical%20conditions%20in%20aged%20care%20%28.pdf?response-content-type=application%2Fpdf&X-Amz-Algorithm=AWS4-HMAC-SHA256&X-Amz-Credential=AKIAYSFKCAWY23RWESRS%2F20220911%2Fus-east-1%2Fs3%2Faws4_request&X-Amz-Date=20220911T061733Z&X-Amz-Expires=604800&X-Amz-SignedHeaders=host&X-Amz-Signature=bde84ac0e5c608f229c6e5eb1f8b5683b12a8ed9dd4d1b6fed0121fdb25147f8	2019	Geriatrics	No	No	Other
**331. Updated guidelines for the management of paracetamol poisoning in Australia and New Zealand**	https://onlinelibrary.wiley.com/doi/abs/10.5694/mja2.50428	2020	Toxicology	No	Yes	Not stated
**332. SOMANZ position paper on the management of nausea and vomiting in pregnancy and hyperemesis gravidarum**	https://regroup-production.s3.amazonaws.com/documents/ReviewReference/350657971/Lowe-2020-SOMANZ%20position%20paper%20on%20the%20managem.pdf?response-content-type=application%2Fpdf&X-Amz-Algorithm=AWS4-HMAC-SHA256&X-Amz-Credential=AKIAYSFKCAWY23RWESRS%2F20220911%2Fus-east-1%2Fs3%2Faws4_request&X-Amz-Date=20220911T061125Z&X-Amz-Expires=604800&X-Amz-SignedHeaders=host&X-Amz-Signature=00674ff13a712fe5ba6274e6e1a02fdf69a9bb6237efcacbc05e5b4ce66ab52a	2019	Women's Health	No	No	Other
**333. Arrhythmogenic right ventricular cardiomyopathy**	https://regroup-production.s3.amazonaws.com/documents/ReviewReference/350660411/2020-Arrhythmogenic%20Right%20Ventricular%20Cardiomy.pdf?response-content-type=application%2Fpdf&X-Amz-Algorithm=AWS4-HMAC-SHA256&X-Amz-Credential=AKIAYSFKCAWY23RWESRS%2F20220911%2Fus-east-1%2Fs3%2Faws4_request&X-Amz-Date=20220911T055926Z&X-Amz-Expires=604800&X-Amz-SignedHeaders=host&X-Amz-Signature=a987fbbcab761899e310beea34186127a251335cce271aaf43a1ca48556653b1	2019	Cardiology	No	No	Not stated

Guideline name	URL	Year	Health topic	NHMRC-approved	GRADE	Funding source
**334. Hypofractionated radiotherapy for early (operable) breast cancer**	http://cancerAustralia.gov.au/system/tdf/guidelines/hypofractionated_radiotherapy_for_early_operable_breast_cancer.pdf?file=1&type=node&id=4041	2015	Cancer	No	Yes	Not stated
**335. International evidence-based guideline for the assessment and management of polycystic ovary syndrome 2018**	https://www.monash.edu/__data/assets/pdf_file/0004/1412644/PCOS_Evidence-Based-Guidelines_20181009.pdf	2018	Women's Health	Yes	Yes	NHMRC
**No named guideline panel members (not included in analysis)**						
**336. Envenomation - jellyfish stings**	https://resus.org.au/?wpfb_dl=41	2010	Intensive care	No	No	Not stated
**337. Clinical practice guideline for the management of women who report decreased fetal movements**	https://sanda.psanz.com.au/assets/Uploads/FINAL-DFM-guideline-Ed1V1-1-16Sept2010.pdf	2010	Women's health	No	No	Other
**338. Guidelines for the treatment of congenital disorders of fibrinogen**	https://www.ahcdo.org.au/documents/item/15	2010	Haematology	No	No	Not stated
**339. Management of specific dysrhythmias in paediatric advanced life support**	https://csds.qld.edu.au/sdc/Provectus/MERT/MER%20CRM/ALS%20Flow%20Charts/newUnit/files/Paed-Management%20of%20Specific%20Arrhythmias%20in%20Paediatric%20Life%20Support.pdf	2010	Intensive care	No	No	Not stated
**340. Cardiopulmonary resuscitation**	https://onlinelibrary.wiley.com/doi/pdfdirect/10.1111/j.1742-6723.2011.01422_9.x	2010	Intensive care	No	No	Not stated
**341. Guidelines for the treatment of inhibitors in haemophilia a and haemophilia B**	https://www.ahcdo.org.au/documents/item/16	2010	Haematology	No	No	Not stated
**342. Medication or fluids for the resuscitation of the newborn infant**	https://onlinelibrary.wiley.com/doi/abs/10.1111/j.1742-6723.2011.01442_14.x	2010	Intensive care	No	No	Not stated
**343. Medications and fluids in paediatric advanced life support**	https://csds.qld.edu.au/sdc/Provectus/MERT/MER%20CRM/ALS%20Flow%20Charts/newUnit/files/Paed-Medications%20and%20Fluids%20in%20Paediatric%20Life%20Suport.pdf	2010	Intensive care	No	No	Not stated
**344. Chest compressions during resuscitation of the newborn infant**	https://onlinelibrary.wiley.com/doi/pdfdirect/10.1111/j.1742-6723.2011.01442_13.x	2010	Intensive care	No	No	Not stated
**345. Medications in adult cardiac arrest**	https://onlinelibrary.wiley.com/doi/pdfdirect/10.1111/j.1742-6723.2011.01422_13.x	2010	Intensive care	No	No	Not stated
**346. Cardiopulmonary resuscitation for advanced life support providers**	https://onlinelibrary.wiley.com/doi/pdfdirect/10.1111/j.1742-6723.2011.01422_9.x	2010	Intensive care	No	No	Not stated
**347. Post-resuscitation therapy in adult advanced life support**	https://onlinelibrary.wiley.com/doi/pdfdirect/10.1111/j.1742-6723.2011.01422_15.x	2010	Intensive care	No	No	Not stated
**348. Electrical therapy for adult advanced life support**	https://onlinelibrary.wiley.com/doi/pdfdirect/10.1111/j.1742-6723.2011.01422_12.x	2010	Intensive care	No	No	Not stated
**349. Circumcision of infant males**	https://ranzcog.edu.au/RANZCOG_SITE/media/RANZCOG-MEDIA/Women%27s%20Health/RACP-Circumcision-of-infant-males-2010.pdf?ext=.pdf	2010	Paediatrics	No	No	Not stated
**350. Equipment and techniques in adult advanced life support**	https://onlinelibrary.wiley.com/doi/pdfdirect/10.1111/j.1742-6723.2011.01422_14.x	2010	Intensive care	No	No	Not stated
**351. Protocols for adult advanced life support**	https://onlinelibrary.wiley.com/doi/pdfdirect/10.1111/j.1742-6723.2011.01422_10.x	2010	Intensive care	No	No	Not stated

Guideline name	URL	Year	Health topic	NHMRC-approved	GRADE	Funding source
**352. Techniques in paediatric advanced life support**	https://onlinelibrary.wiley.com/doi/pdfdirect/10.1111/j.1742-6723.2011.01442_6.x	2010	Intensive care	No	No	Not stated
**353. resuscitation of the newborn infant in special circumstances**	https://onlinelibrary.wiley.com/doi/pdfdirect/10.1111/j.1742-6723.2011.01442_15.x	2010	Intensive care	No	No	Federal government
**354. Introduction to paediatric advanced life support**	https://onlinelibrary.wiley.com/doi/pdfdirect/10.1111/j.1742-6723.2011.01442_1.x	2010	Intensive care	No	No	Not stated
**355. Advanced life support for infants and children. Diagnosis and management**	https://onlinelibrary.wiley.com/doi/pdfdirect/10.1111/j.1742-6723.2011.01442_2.x	2010	Intensive care	No	No	Not stated
**356. Therapeutic hypothermia after cardiac arrest**	https://onlinelibrary.wiley.com/doi/pdfdirect/10.1111/j.1742-6723.2011.01422_16.x	2010	Intensive care	No	No	Not stated
**357. Introduction to resuscitation of the newborn infant**	https://onlinelibrary.wiley.com/doi/pdfdirect/10.1111/j.1742-6723.2011.01442_8.x	2010	Intensive care	No	No	Not stated
**358. After the resuscitation of a newborn infant**	https://onlinelibrary.wiley.com/doi/pdfdirect/10.1111/j.1742-6723.2011.01442_16.x	2010	Intensive care	No	No	Not stated
**359. Guideline for the management of patients with haemophilia undergoing surgical procedures**	https://www.ahcdo.org.au/documents/item/13	2010	Haematology	No	No	Not stated
**360. Resuscitation of divers who have used compressed gas**	http://www.resus.org.nz/assets/Guidelines/First-Aid/guideline-9-3-5-nov11.pdf	2011	Intensive care	No	No	Not stated
**361. Clinical practice guidelines for surveillance colonoscopy - in adenoma follow-up; following curative resection of colorectal cancer; and for cancer surveillance in inflammatory bowel disease (December 2011)**	https://wiki.cancer.org.au/Australia/Guidelines:Colorectal_cancer/Colonoscopy_surveillance	2011	Cancer	Yes	No	Federal government
**362. Gastro-oesophageal reflux disease in adults (5th edition)**	https://www.gesa.org.au/public/13/files/Education%20%26%20Resources/Clinical%20Practice%20Resources/GORD/Reflux_Disease.pdf	2011	Gastroenterology	No	No	Other
**363. Guidelines for management of factor VII deficiency**	https://www.ahcdo.org.au/documents/item/10	2010	Haematology	No	No	Not stated
**364. Airway management and mask ventilation of the newborn infant**	https://onlinelibrary.wiley.com/doi/pdfdirect/10.1111/j.1742-6723.2011.01442_11.x	2010	Intensive care	No	No	Not stated
**365. Tracheal intubation and ventilation of the newborn infant**	https://onlinelibrary.wiley.com/doi/pdfdirect/10.1111/j.1742-6723.2011.01442_12.x	2010	Intensive care	No	No	Not stated
**366. Guidelines for prevention of transfusion-associated graft-versus-host disease (TA-GVHD)**	https://www.nzblood.co.nz/assets/Transfusion-Medicine/PDFs/First-Edition-January-2011-Guidelines-PreventionofTA-GVHD.pdf.pdf	2011	Haematology	No	No	Not stated
**367. Australian clinical guidelines for psychosis. A brief summary for practitioners**	https://www.ranzcp.org/files/resources/college_statements/clinician/cpg/early-psychosis-cpg.aspx	2011	Psychiatry	No	No	Not stated
**368. Guidelines for the diagnosis and management of familial long QT syndrome**	https://www.csanz.edu.au/documents/guidelines/clinical_practice/Familial_Long_QT_Syndrome.pdf	2011	Cardiology	No	No	Not stated
**369. Guidelines for the diagnosis and management of Marfan syndrome**	https://csanz.edu.au/documents/guidelines/clinical_practice/Marfan_Syndrome.pdf	2011	Cardiology	No	No	Not stated
**370. Precordial thump and fist pacing**	https://onlinelibrary.wiley.com/doi/pdfdirect/10.1111/j.1742-6723.2011.01422_11.x	2010	Intensive care	No	No	Not stated

Guideline name	URL	Year	Health topic	NHMRC-approved	GRADE	Funding source
**371. Acute coronary syndromes - presentation with ACS**	https://onlinelibrary.wiley.com/doi/pdfdirect/10.1111/j.1742-6723.2011.01422_18.x	2011	Intensive care	No	No	Not stated
**372. Australian and New Zealand society for geriatric medicine position statement 19. Obesity and the older person**	https://anzsgm.org/wp-content/uploads/2019/02/ObesityandtheOlderPerson11Sept113.pdf	2011	Cardiology	No	No	Not stated
**373. Automated external defibrillation (AED) in basic life support (BLs)**	https://www.broomesearescue.org.au/member_info/Automated%20External%20Defibrillation%20(AED)%20Guidelines.pdf	2011	Intensive care	No	No	Not stated
**374. Resuscitation in special circumstances**	https://static1.squarespace.com/static/5a0d48dbe9bfdfcb922bc863/t/5ad3212888251b90ed417860/1523786028509/NZRCguideline-11-10-nov2011.pdf	2011	Intensive care	No	No	Not stated
**375. Acute coronary syndromes - reperfusion strategy**	https://onlinelibrary.wiley.com/doi/pdfdirect/10.1111/j.1742-6723.2011.01422_20.x	2011	Intensive care	No	No	Not stated
**376. Emergency management of a victim who has been poisoned**	https://www.nzrc.org.nz/assets/Guidelines/First-Aid/guideline-9-5-1-july11.pdf	2011	Intensive care	No	No	Not stated
**377. Guidelines for the diagnosis and management of Brugada syndrome**	http://csanz.edu.au/documents/guidelines/clinical_practice/Brugada_Syndrome.pdf	2011	Cardiology	No	No	Not stated
**378. Envenomation - Australian snake bite**	https://icmwk.com/wp-content/uploads/2015/06/ARC-snakes.pdf	2011	Intensive care	No	No	Not stated
**379. Envenomation - pressure immobilisation technique**	https://survive-student-resource.austererisk.com/environmental/anzcor_9_4_8_pressure_immobilisation.html	2011	Intensive care	No	No	Not stated
**380. Anaphylaxis - first aid management**	http://www.nationalfirstaid.com.au/wp-content/uploads/Anaphylaxs-First-Aid-Management-Guideline-9-2-7-July12.pdf	2012	Intensive care	No	No	Not stated
**381. Investigating symptoms of lung cancer - a guide for GPs**	https://www.cancerAustralia.gov.au/sites/default/files/publications/lung-cancer-gp-guide-2012_509ae0cdd2e36.pdf	2012	Cancer	No	No	Not stated
**382. Allergic rhinitis and asthma. An information paper for health professionals**	https://d8z57tiamduo7.cloudfront.net/resources/NAC-Managing-Allergic-Rhinitis-2020.pdf	2012	Respiratory	No	No	Other
**383. Asthma and complementary therapies. An information paper for health professionals**	https://www.researchgate.net/publication/268280311_Asthma_and_Complementary_Therapies_An_information_paper_for_health_professionals/link/551a6e6a0cf26cbb81a2d49d/download	2012	Respiratory	No	No	Federal government
**384. Recognition and first aid management of the unconscious victim**		2012	Intensive care	No	No	Not stated
**385. General practitioners and hepatitis C**	https://www.aph.gov.au/DocumentStore.ashx?id=18b58c4b-7aa3-4ad3-98f3-66cb50fbcdb7&subId=303939	2012	Infectious diseases	No	No	Federal government
**386. Primary care providers and hepatitis C**	https://www.aph.gov.au/DocumentStore.ashx?id=18b58c4b-7aa3-4ad3-98f3-66cb50fbcdb7&subId=303939	2012	Infectious diseases	No	No	Federal government
**387. Hepatitis C: your crucial role as a primary health care nurse**	https://www.aph.gov.au/DocumentStore.ashx?id=4799afa2-cddb-46cd-a393-f23719622ef8&subId=303939	2012	Infectious diseases	No	No	Federal government
**388. Acute coronary syndromes - initial medical therapy**	https://www.saudedireta.com.br/docsupload/1425662722guideline-14-2-july12.pdf	2012	Intensive care	No	No	Not stated
**389. Guidelines for the diagnosis and management of familial dilated cardiomyopathy**	https://www.csanz.edu.au/wp-content/uploads/2013/12/Familial_Dilated_Cardiomyopathy_2013.pdf	2013	Cardiology	No	No	Not stated

Guideline name	URL	Year	Health topic	NHMRC-approved	GRADE	Funding source
**390. Emergency management of a crushed victim**	https://www.nzrc.org.nz/assets/Guidelines/First-Aid/guideline-9-1-7-march2013.pdf	2013	Intensive care	No	No	Not stated
**391. Asthma and healthy living. An information paper for health professionals**	https://d8z57tiamduo7.cloudfront.net/resources/asthma-healthy-living-hp.pdf	2013	Respiratory	No	No	Federal government
**392. The BCG vaccine: information and recommendations for use in Australia**	https://www1.health.gov.au/internet/main/publishing.nsf/Content/cda-cdi3701-pdf-cnt.htm/$FILE/cdi3701h.pdf	2012	Infectious diseases	No	No	Not stated
**393. Envenomation - blue-ringed octopus and cone shell**	https://resus.org.au/guidelines/	2014	Intensive care	No	No	Not stated
**394. Motor neurone disease. Aspects of care for the primary health care team**	https://www.mndcare.net.au/Overview/MNDcare-approach/Information-and-discussion/For-health-and-community-care-professionals/MND-Australia-2014/MND-Aspects-of-care-for-the-primary-health-car-(1).aspx	2014	Neurology	No	No	Not stated
**395. Envenomation - fish stings**	https://resus.org.au/guidelines/	2014	Intensive care	No	No	Not stated
**396. First aid management of a seizure**	https://www.nzrc.org.nz/assets/Guidelines/First-Aid/Guideline-9-2-4-Nov14.pdf	2014	Intensive care	No	No	Not stated
**397. Guidelines on sedation and/or analgesia for diagnostic and interventional medical, dental or surgical procedures**	https://www.anzca.edu.au/getattachment/c64aef58-e188-494a-b471-3c07b7149f0c/PS09-Guideline-on-sedation-and-or-analgesia-for-diagnostic-and-interventional-medical,-dental-or-surgical-procedures	2014	Intensive care	No	No	Not stated
**398. Envenomation - spider bite**	https://www.spiders.com.au/Funnel%20Web%20and%20Mouse%20Spider%20Bites%20-%20Australian%20Resuscitation%20Council.pdf	2014	Intensive care	No	No	Not stated
**399. Resuscitation of the drowning victim**	https://resus.org.au/?wpfb_dl=33	2014	Intensive care	No	No	Not stated
**400. Recommendations for the management of early breast cancer in women with an identified BRCA1 or BRCA2 gene mutation or at high risk of a gene mutation**	https://www.cancerAustralia.gov.au/publications-and-resources/cancer-Australia-publications/recommendations-management-early-breast-cancer-women-identified-brca1-or-brca2-gene-mutation-or-high	2015	Cancer	No	No	Federal government
**401. ASCIA guidelines - infant feeding and allergy prevention**	https://regroup-production.s3.amazonaws.com/documents/ReviewReference/350660211/2016-ASCIA%20Guidelines%20-%20Infant%20feeding%20and%20all.pdf?response-content-type=application%2Fpdf&X-Amz-Algorithm=AWS4-HMAC-SHA256&X-Amz-Credential=AKIAYSFKCAWY23RWESRS%2F20220911%2Fus-east-1%2Fs3%2Faws4_request&X-Amz-Date=20220911T133131Z&X-Amz-Expires=604800&X-Amz-SignedHeaders=host&X-Amz-Signature=0eac8cc9613cca73b77b61a1800c832738c351af6352351f42683ab7fc687ed6	2016	Immunology	No	No	None
**402. Familial long QT syndrome**	https://regroup-production.s3.amazonaws.com/documents/ReviewReference/350660220/2016-Familial%20Long%20QT%20Syndrome.pdf?response-content-type=application%2Fpdf&X-Amz-Algorithm=AWS4-HMAC-SHA256&X-Amz-Credential=AKIAYSFKCAWY23RWESRS%2F20220911%2Fus-east-1%2Fs3%2Faws4_request&X-Amz-Date=20220911T132620Z&X-Amz-Expires=604800&X-Amz-SignedHeaders=host&X-Amz-Signature=ef712e213fe0167515e77dce39b4666b6804fb34e0ae4035a16620de35baa5bf	2016	Cardiology	No	No	Not stated

Guideline name	URL	Year	Health topic	NHMRC-approved	GRADE	Funding source
**403. Catecholaminergic polymorphic ventricular tachycardia**	https://regroup-production.s3.amazonaws.com/documents/ReviewReference/350659648/2016-Catecholaminergic%20Polymorphic%20Ventricular.pdf?response-content-type=application%2Fpdf&X-Amz-Algorithm=AWS4-HMAC-SHA256&X-Amz-Credential=AKIAYSFKCAWY23RWESRS%2F20220911%2Fus-east-1%2Fs3%2Faws4_request&X-Amz-Date=20220911T132430Z&X-Amz-Expires=604800&X-Amz-SignedHeaders=host&X-Amz-Signature=42a4ea1ad2b1eadbeab5afa9d015fb522d3f063f9e07592d6232695afc1fee11	2016	Cardiology	No	No	Not stated
**404. ASCIA guidelines - vaccination of the egg allergic individual**	https://regroup-production.s3.amazonaws.com/documents/ReviewReference/350659906/2017-ASCIA%20Guidelines%20-%20vaccination%20of%20the%20egg.pdf?response-content-type=application%2Fpdf&X-Amz-Algorithm=AWS4-HMAC-SHA256&X-Amz-Credential=AKIAYSFKCAWY23RWESRS%2F20220911%2Fus-east-1%2Fs3%2Faws4_request&X-Amz-Date=20220911T131739Z&X-Amz-Expires=604800&X-Amz-SignedHeaders=host&X-Amz-Signature=753755bf00383d50bc25edf9e59a7a1d345d823e89eff2eed34290ec9c3087df	2017	Immunology	No	No	None
**405. ASCIA clinical update infant feeding and allergy prevention**	https://regroup-production.s3.amazonaws.com/documents/ReviewReference/350660179/2018-ASCIA%20Clinical%20Update%20Infant%20Feeding%20and.pdf?response-content-type=application%2Fpdf&X-Amz-Algorithm=AWS4-HMAC-SHA256&X-Amz-Credential=AKIAYSFKCAWY23RWESRS%2F20220911%2Fus-east-1%2Fs3%2Faws4_request&X-Amz-Date=20220911T063328Z&X-Amz-Expires=604800&X-Amz-SignedHeaders=host&X-Amz-Signature=fec13227ce233a91ebf4b5864145740cc701e0c365f78a5b5e6650cc5179e4b2	2018	Immunology	No	No	None
**406. Assessment of the newborn infant**	https://onlinelibrary.wiley.com/doi/epdf/10.1111/j.1742-6723.2011.01442_10.x	2010	Intensive care	No	No	Not stated

Table 2. Overall proportions of guideline panel members who were women in 335 guidelines, by health topic

		Proportion of guideline panel members		
**Health topic**	**Number of guidelines**	**< 40%**	**40-60%**	**>60%**
Cancer	50	22 (44%)	14 (28%)	14 (28%)
Cardiology	39	31 (79%)	4 (10%)	4 (10%)
Nephrology	27	18 (67%)	8 (30%)	1 (4%)
Endocrinology	23	17 (74%)	1 (4%)	5 (22%)
Mental health	22	12 (55%)	7 (32%)	3 (14%)
Infectious diseases	21	10 (48%)	9 (43%)	2 (10%)
Women's health	17	2 (12%)	3 (18%)	12 (71%)
Pathology	17	14 (83%)	1 (6%)	2 (12%)
Paediatric medicine	13	4 (31%)	3 (23%)	6 (46%)
Respiratory	13	7 (54%)	4 (31%)	2 (15%)
Haematology	13	10 (77%)	3 (23%)	0
General practice	8	3 (38%)	3 (38%)	2 (25%)
Immunology	7	2 (29%)	3 (43%)	2 (29%)
Intensive care	7	5 (71%)	1 (14%)	1 (14%)
Public health	7	1 (14%)	4 (57%)	2 (29%)
Neurology	6	0	2 (33%)	4 (67%)
Gastroenterology	5	3 (60%)	1 (20%)	1 (20%)
Nutrition	5	1 (20%)	1 (20%)	3 (60%)
Anaesthetics	4	3 (75%)	1 (25%)	0
Geriatric medicine	3	0	2 (67%)	1 (33%)
Urology	3	2 (67%)	0	1 (33%)
Dermatology	3	2 (67%)	0	1 (33%)
Orthopaedics	3	0	3 (100%)	0
Gambling and addiction	3	1 (33%)	2 (67%)	0
Optometry	1	0	1 (100%)	0
Toxicology	2	2 (100%)	0	0
Exercise medicine	2	0	0	2 (100%)
Osteoporosis	2	0	0	2 (100%)
Rheumatology	2	2 (100%)	0	0
Palliative care	2	2 (100%)	0	0
Emergency medicine	1	1 (100%)	0	0
Sexual health	1	0	1 (100%)	0
Dentistry	1	1 (100%)	0	0
Aviation medicine	1	1 (100%)	0	0
Surgery	1	1 (100%)	0	0

Table 3. The 335 included guidelines: numbers by year of publication, and numbers of female guideline panel chairs and members

Year	Number of guidelines	Women guideline panel chairs	Women guideline panel members
**2010**	29	8/23 (35%)	127/367 (35%)
**2011**	44	23/62 (37%)	288/572 (50%)
**2012**	44	19/52 (37%)	223/574 (39%)
**2013**	35	24/59 (41%)	133/409 (33%)
**2014**	51	19/41 (46%)	206/549 (38%)
**2015**	14	4/11 (36%)	65/223 (29%)
**2016**	21	7/35 (20%)	118/313 (38%)
**2017**	21	38/62 (62%)	297/536 (55%)
**2018**	26	20/44 (45%)	166/388 (43%)
**2019**	22	17/42 (40%)	116/225 (45%)
**2020**	28	36/81 (44%)	332/853 (39%)
